# Resource Use and Care Quality Differences Among Medicare Beneficiaries Undergoing Chemotherapy

**DOI:** 10.1001/jamanetworkopen.2024.34707

**Published:** 2024-09-20

**Authors:** Yamini Kalidindi, Jeah Jung, Roger Feldman, Caroline Carlin, Ge Song, Aaron Mitchell

**Affiliations:** 1 McDermott+ Consulting, Washington, DC; 2 Department of Health Administration and Policy, College of Public Health, George Mason University, Fairfax, Virginia; 3 Division of Health Policy and Management, School of Public Health, University of Minnesota, Minneapolis; 4 Department of Family Medicine and Community Health, University of Minnesota, Minneapolis; 5 Memorial Sloan Kettering Cancer Center, New York, New York

## Abstract

**Importance:**

Medicare Advantage (MA) has grown significantly over the last decade; however, MA’s performance for patients with serious conditions, such as cancer, remains unclear.

**Objective:**

To compare resource use and care quality between MA and traditional Medicare (TM) beneficiaries undergoing cancer chemotherapy.

**Design, Setting, and Participants:**

This cohort study used TM claims and MA encounter records from January 2015 to December 2019. Participants were MA and TM beneficiaries who initiated cancer chemotherapy between January 2016 and July 2019. Inverse probability of treatment weighting balanced characteristics between MA and TM beneficiaries, and regression estimation was used. The analysis was conducted between August 2023 and May 2024.

**Exposure:**

Chemotherapy initiation after a 1-year washout period.

**Main Outcomes and Measures:**

Resource use and care quality were measured during a 6-month period following chemotherapy initiation. Resource use was measured using standardized prices for services in both MA and TM, covering hospital inpatient services, outpatient care, Part D drugs, and hospice services. Chemotherapy utilization was examined for Part B chemotherapy, Part B supportive drugs, and Part D chemotherapy. Quality measures included chemotherapy-related emergency department (ED) visits and hospitalizations, avoidable ED visits, preventable hospitalizations during the 6-month episode, and survival days up to 18 months from chemotherapy initiation.

**Results:**

The study comprised 122 966 MA enrollees contributing to 125 518 episodes (mean [SD] age, 73.2 [7.6] years; 68 479 [54.6%] female; 8900 [7.1%] Hispanic, 18 423 [14.7%] non-Hispanic Black, and 94 817 [75.5%] non-Hispanic White participants) and 274 666 TM beneficiaries, contributing 282 200 episodes (mean [SD] age, 73.1 [8.4] years; 155 709 [55.2%] female; 10 666 [3.8%] Hispanic, 22 088 [7.8%] non-Hispanic Black, and 241 113 [85.4%] non-Hispanic White participants). Adjusted total resource use per enrollee during the 6-month episode was $8498 (95% CI, $8178 to $8817) lower in MA than TM ($61 004 vs $69 502). Part B chemotherapy resource use accounted for most of the difference in total resource use, with MA enrollees having $4765 (95% CI, $4547 to $4982) lower use than TM beneficiaries. Lower resource use for Part B chemotherapy in MA was associated with 1.03 fewer chemotherapy visits (95% CI, 0.99 to 1.06 visits) and $277 (95% CI, $183 to $272) less expensive chemotherapy per visit. Findings on quality were mixed, but importantly, survival did not differ between MA and TM patients who initiated chemotherapy.

**Conclusions and Relevance:**

In this cohort study of Medicare beneficiaries with cancer undergoing chemotherapy, MA enrollment was associated with lower resource use but not shorter survival.

## Key Points

**Question:**

Are there differences in resource use and quality of care between patients with cancer undergoing chemotherapy who are enrolled in Medicare Advantage (MA) vs traditional Medicare (TM)?

**Findings:**

In this cohort study of 122 966 MA and 274 666 TM beneficiaries receiving chemotherapy, MA enrollment was associated with $8498 lower adjusted total resource use during a 6-month chemotherapy episode, mostly due to lower resource use for chemotherapy. The findings on quality of care were mixed, and there was no difference in survival among MA and TM beneficiaries who initiated chemotherapy.

**Meaning:**

In this study, MA enrollment was associated with lower resource use but not shorter survival among Medicare beneficiaries receiving chemotherapy.

## Teaser

This cohort study examines whether there are differences in resource use or care quality between patients with cancer undergoing chemotherapy who are enrolled in Medicare Advantage vs traditional Medicare.

## Introduction

A significant shift in health care enrollment is occurring in the US Medicare program. Private Medicare Advantage (MA) plans enrolled more than half of the Medicare population in 2023,^1^ and MA enrollment is projected to reach 61% by 2032.^2^ While MA plans offer enhanced coverage, such as lower cost sharing and supplemental benefits, increased MA enrollment raises important questions regarding how MA may impact health care spending and care quality.

Prior studies reported that health care spending per enrollee in MA was 9% to 36% lower than in traditional Medicare (TM).^3,4,5,6^ They also found that MA plans reduced spending by lowering utilization of high-cost services, such as hospitalizations. These findings are based on comparisons of total costs and service utilization in broad populations, as opposed to condition-specific outcomes.

Other prior work on MA-TM differences in service utilization focused on specific conditions managed in the primary care setting, such as diabetes or cardiovascular diseases.^7,8,9^ Evidence is limited on MA performance for patients with serious chronic conditions, such as cancer, which requires specialist care and expensive treatments.

Expectations are mixed about whether MA patients may have different patterns of resource use for specialist-focused conditions. Some argue that MA reduces resource use primarily for conditions for which primary care management helps prevent resource-intensive services but not for conditions requiring unavoidable high-cost services or specialist interventions.^10^ Substantial cost savings might be achievable by navigating patients toward lower-cost specialists or services.^11^ However, some express concern that MA plans may restrict access to medically necessary high-cost services.^12^

A recent analysis found that MA enrollment was associated with lower costs for inpatient cancer surgery, but the study also found potential drawbacks to MA enrollment, such as reduced access to high-volume hospitals and increased 30-day mortality rates.^13^ However, this prior analysis was limited to patients receiving cancer surgery in one state and did not examine the association of MA enrollment with cancer treatment costs. The financial burden of cancer care is substantial, estimated at $208.9 billion in 2020.^14^ A considerable portion of cancer care cost stems from chemotherapy, as new and high-priced treatments become available.^15^ Given that MA plans use various utilization management techniques, such as prior authorization, step therapies, and quantity limits^16,17^ while also promoting care coordination and quality incentives,^18^ there is a pressing need to examine whether patterns of chemotherapy-related costs and quality for patients with cancer differ between MA and TM.

To address this gap, we used national Medicare data to compare resource use and care quality between MA and TM beneficiaries with cancer, focusing on those receiving chemotherapy. We also examine sources of MA-TM differences in resource use for chemotherapy: treatment intensity and costliness of chemotherapeutic agent. To our knowledge, our study provides the first information about the association between MA enrollment and treatment patterns for patients with cancer receiving chemotherapy.

## Methods

The George Mason University institutional review board determined this study did not involve human participants and waived the requirement for informed consent. This study followed the Strengthening the Reporting of Observational Studies in Epidemiology (STROBE) reporting guideline.

### Data

We used 100% national MA encounter data and TM claims data from January 1, 2015, to December 31, 2019. Following prior work, we used MA encounter data only from contracts with highly complete data.^19^ The 2015 data were used only to identify patients with a 1-year washout period (no chemotherapy for 1 year prior to identification) and to construct health risk scores based on prior-year claims.

The Medicare Beneficiary Summary File supplied information on death dates and patient demographic characteristics. The Area Health Resources File provided area-level health care environment variables, and the American Community Survey supplied zip code–level socioeconomic variables.

### Study Population

The study population comprised Medicare beneficiaries who had 1 of 7 cancers: breast cancer, chronic leukemia, colorectal or small intestine cancer, lung cancer, lymphoma, multiple myeloma, and prostate cancer, identified following the Enhancing Oncology Model,^20^ and who initiated chemotherapy between January 1, 2016, and July 31, 2019, after at least a 1-year washout period with no chemotherapy, with coverage observed through the end of the 1-year observation period. We use the term chemotherapy to include cytotoxic, targeted, and immunotherapy agents. We excluded patients who received only hormonal therapy. To assign cancer types and identify chemotherapy, we followed the approaches used in the oncology care model by the Centers for Medicare & Medicaid Services (CMS).^20,21^ Further details on these approaches are included in the eMethods in Supplement 1.

We defined the index date as the first chemotherapy date after the washout period and a chemotherapy episode as the 6-month period from the index date, or until death if it occurred earlier for our primary analyses. We required the beneficiary to have continuous Parts A, B, or D coverage during the washout period and the 1-year observation period following chemotherapy initiation; stay in one program (either MA or TM) during the entire chemotherapy washout period and 1-year period following chemotherapy initiation; not have end-stage kidney disease; and reside in the 50 US states or the District of Columbia. We imposed a 1-year coverage restriction after chemotherapy initiation because one of our sensitivity analyses used a 1-year episode and holding the coverage constant allowed for better comparison of the primary and sensitivity analyses.

### Outcomes

We measured outcomes during the 6-month chemotherapy episode. We calculated resource use by applying standardized prices to services delivered in both MA and TM. Thus, differences in resource use represent differences in service utilization. Following prior work,^19,22^ we used mean TM payments by service type as the standardized prices. We measured total resource use and resource use separately for the following service types: hospital inpatient services, outpatient care, Part D prescription drugs, and hospice services. We identified all hospital stays for MA patients by supplementing missing hospital stays in MA encounter records with hospital stays from Medicare Provider Analysis and Review files.^19^ We did not examine resource use for skilled nursing facilities and home health care agencies because of incomplete encounter data for those services.^19^

We examined resource use separately for each of the following chemotherapy services: Part B chemotherapy, Part B supportive care drugs, and Part D chemotherapy (list of chemotherapy and supportive care drugs is provided in eTable 7 in Supplement 1). Furthermore, we examined the sources of differences in resource use for Part B chemotherapy stemming from treatment intensity vs costliness of the selected chemotherapy agents. We measured intensity by Part B chemotherapy visit-days (hereafter visits) per 6-month episode and use of costly chemotherapy by resource use per Part B chemotherapy visit.

We computed 2 chemotherapy-related quality measures, chemotherapy-related ED visits and chemotherapy-related hospitalizations, used by CMS and endorsed by the National Quality Forum.^23^ Additionally, we computed 2 measures of adverse health events—avoidable ED visits^24,25^ and preventable hospitalizations^26^—which could be avoided through effective care management in the ambulatory care setting. Finally, we measured survival days from the index date through 18 months. Survival differences larger than 30 days are generally considered to be clinically meaningful.^27^ Further details regarding the quality measures are provided in the eMethods in Supplement 1.

### Explanatory Variables

The key independent variable was MA enrollment. The control variables included age, gender, race and ethnicity, dual eligibility for Medicare and Medicaid, cancer metastasis, claims-based frailty index,^28^ and a summary health risk score measured by hierarchical condition category scores. Race and ethnicity were recorded in the Master Beneficiary Summary File (categorized as Hispanic, non-Hispanic Black, non-Hispanic White, and other [includes American Indian or Alaska Native, Asian or Pacific Islander, and unknown]). Similar to the other covariates included as controls, race and ethnicity have often been shown to be strongly correlated with health care utilization. We also used an indicator for zip code–level rurality; zip code–level socioeconomic variables including the percentages of college-educated residents, of households living under the federal poverty line, and of people speaking English only; and county-level variables including hospital beds per 1000 residents, physicians per 1000 residents, and skilled nursing facility beds per 1000 residents. Further details on covariates are provided in the eMethods in Supplement 1.

### Statistical Analysis

We compared the baseline characteristics between MA and TM patients by calculating means and *t* tests for continuous variables, and percentages and Pearson χ^2^ tests for categorical variables. We used inverse probability of treatment weighting to balance observed covariate distributions between MA and TM. We estimated weighted linear regressions with county fixed effects to account for time-constant county factors. Using the regression results, we calculated the risk-adjusted outcomes for each program (MA and TM), setting all covariates at their mean values. Statistical significance was assessed at *P* < .05 from 2-sided tests. Further details on methodology are in the eMethods in Supplement 1.

We also conducted subgroup analyses by cancer type and MA plan type, defined as health maintenance organizations (HMOs) or preferred provider organizations (PPOs). The total population of TM beneficiaries was used for the analysis by plan type.

We performed 3 sensitivity checks. First, we ran regressions using 1-year chemotherapy episodes instead of 6-month episodes to assess the stability of results over a longer episode duration. Second, 4.4% of the study sample had more than 1 chemotherapy episode during the study period with no treatment for at least 1 year between the episodes. We performed the analysis including only the first episode for each beneficiary. Third, we estimated a generalized linear model for resource use and used logit analyses for the quality outcomes. We used SAS version 9.4 (SAS Institute) and Stata version 17 (StataCorp) to conduct analyses.

## Results

### Sample Characteristics

The study comprised 122 966 MA enrollees contributing to 125 518 episodes (mean [SD] age, 73.2 [7.6] years; 68 479 [54.6%] female; 8900 [7.1%] Hispanic, 18 423 [14.7%] non-Hispanic Black, and 94 817 [75.5%] non-Hispanic White participants) and 274 666 TM beneficiaries, contributing 282 200 episodes (mean [SD] age, 73.1 [8.4] years; 155 709 [55.2%] female; 10 666 [3.8%] Hispanic, 22 088 [7.8%] non-Hispanic Black, and 241 113 [85.4%] non-Hispanic White participants). In the unweighted populations (Table 1), TM patients were more likely to be White, have dual eligibility, and be located in zip codes with larger percentages of college-educated people compared with MA patients. The balance between the MA and TM populations improved significantly when we used inverse probability of treatment weights (Table 1).

**Table 1.** Population Characteristics

Characteristic	Unweighted population			Weighted population^a^		
	Episodes, No. (%)		*P* value^b^	Episodes, %		*P* value^b^
	MA	TM		MA	TM	
Gender						
Female	68 479 (54.6)	155 709 (55.2)	<.001	55.1	55.0	.79
Male	57 039 (45.4)	126 491 (44.8)	NA	44.9	45.0	NA
Age, y						
<64	10 977 (8.7)	26 217 (9.3)	NA	9.0	9.1	NA
65 to <70	27 600 (22.0)	61 679 (21.9)	<.001	21.9	21.9	.73
70 to <75	34 737 (27.7)	73 767 (26.1)	<.001	26.7	26.6	.64
75 to <80	27 637 (22.0)	60 332 (21.4)	<.001	21.6	21.6	.79
80 to <85	15 900 (12.7)	36 903 (13.1)	.05	12.9	13.0	.84
≥85	8667 (6.9)	23 302 (8.3)	<.001	7.8	7.8	.62
Race						
Asian or Pacific Islander	2388 (1.9)	5565 (2.0)	<.001	1.9	1.9	.31
Hispanic	8900 (7.1)	10 666 (3.8)	<.001	4.7	4.7	.77
Non-Hispanic Black	18 423 (14.7)	22 088 (7.8)	<.001	10.0	10.0	.79
Non-Hispanic White	94 817 (75.5)	241 113 (85.4)	NA	82.5	82.4	NA
Other^c^	990 (0.8)	2768 (1.0)	.01	0.9	0.9	.28
Cancer type						
Breast	23 553 (18.8)	48 813 (17.3)	<.001	17.7	17.7	.75
Colorectal	16 131 (12.9)	40 182 (14.2)	<.001	13.7	13.8	.38
Leukemia	7375 (5.9)	18 775 (6.7)	<.001	6.4	6.4	.95
Lung	40 064 (31.9)	86 955 (30.8)	<.001	31.3	31.2	.44
Lymphoma	20 798 (16.6)	51 267 (18.2)	<.001	17.7	17.7	.79
Myeloma	10 415 (8.3)	22 665 (8.0)	<.001	8.1	8.1	.98
Prostate	7182 (5.7)	13 546 (4.8)	<.001	5.1	5.8	.85
Dual eligible	21 206 (16.9)	57 341 (20.3)	<.001	19.6	19.3	.05
Metastatic cancer	48 451 (38.6)	99 984 (35.4)	<.001	36.5	36.4	.65
Frailty index, mean (SD)	0.184 (0.06)	0.188 (0.07)	<.001	0.187 (0.06)	0.187 (0.07)	.37
HCC score						
0 to <1	10 199 (8.1)	22 208 (7.9)	NA	7.9	7.9	NA
1 to <2	29 816 (23.8)	66 699 (23.6)	.05	23.6	23.7	.72
2 to <3	52 014 (41.4)	117 869 (41.8)	<.001	41.7	41.7	.93
≥4	33 489 (26.7)	75 424 (26.7)	.01	26.8	26.7	.67
County-level health care resources, mean (SD)						
Beds/1000, No.	3.1 (2.1)	3.0 (2.3)	<.001	3.0 (2.2)	3.0 (2.3)	.16
Doctors/1000, No.	3.3 (2.3)	3.2 (2.4)	<.001	3.2 (2.3)	3.2 (2.4)	.23
SNF beds/1000, No.	5.6 (2.9)	6.1 (3.4)	<.001	5.9 (3.4)	5.9 (3.2)	.13
Rural	22 342 (17.8)	75 713 (26.8)	<.001	24.0	24.0	.96
Zip code demographics, mean (SD), %						
College educated	29.2 (15.4)	31.9 (17.3)	<.001	30.9 (16.5)	31.1 (16.9)	.05
Speaking English only	82.8 (18.1)	84.2 (16.5)	<.001	84.0 (16.9)	83.9 (16.9)	<.001
Under federal poverty level	13.8 (8.3)	12.6 (7.9)	<.001	13.0 (7.9)	13.0 (8.1)	.69
Episodes, No.	125 518	282 200	NA	NA	NA	NA
Beneficiaries, No.	122 966	274 666	NA	NA	NA	NA

Abbreviations: HCC, Hierarchical Condition Category; MA, Medicare Advantage; NA, not applicable; SNF, skilled nursing facility; TM, traditional Medicare.

^a^ Inverse probability of treatment weighting was used to balance the MA and TM populations for characteristics.

^b^ *t* Tests were used for continuous variables and Pearson χ^2^ tests for categorical variables.

^c^ Other race is a category defined as American Indian or Alaska Native, Asian or Pacific Islander, or unknown in the Master Beneficiary Summary File.

### MA Enrollment and Resource Use

Table 2 presents regression estimates of the association between MA enrollment and study outcomes during the 6-month chemotherapy episode. Risk-adjusted total resource use was $8498 (95% CI, $8178-$8817) lower among MA enrollees ($61 004) compared with TM ($69 502). Resource use for outpatient care was responsible for a substantial portion of the difference in total resource use, with MA enrollees having $8248 (95% CI, $7972-$8524) lower resource use for outpatient care than TM beneficiaries ($38 360 vs $46 608). We report the results for all covariates from the regression analysis for total resource use in eTable 1 in Supplement 1.

**Table 2.** Regression Results and Adjusted Outcomes

Outcome	Regression results, coefficient (95% CI)^a^	Adjusted means (SE)	
		MA	TM
Resource use, US $			
Total resource use	−8498 (−8817 to −8178)	61 004 (132.86)	69 502 (81.56)
By service type			
Hospital inpatient services	−700 (−828 to −573)	9244 (51.33)	9944 (36.15)
Outpatient care	−8248 (−8524 to −7972)	38 360 (114.51)	46 608 (69.97)
Prescription drugs	403 (235 to 572)	12 842 (68.79)	12 439 (46.35)
Hospice services	48 (29 to 66)	558 (7.83)	511 (4.75)
Chemotherapy services^b^			
Chemotherapy services, US $			
Part B chemotherapy	−4765 (−4982 to −4547)	18 893 (87.72)	23 658 (60.10)
Part B chemotherapy-supportive drugs	−415 (−435 to −396)	2222 (7.82)	2637 (5.06)
Part D chemotherapy	337 (175 to 498)	10 605 (66.02)	10 269 (44.42)
Sources of differences in part B chemotherapy			
Chemotherapy visits per episode	−1.03 (−1.06 to −0.99)	5.92 (0.01)	6.94 (0.01)
Resource use/chemotherapy visit, US $	−227 (−272 to −183)	3377 (18.79)	3604 (9.30)
Quality of care			
Chemotherapy-related ED visits, percentage points^c^	−2.91 (−3.22 to −2.60)	23.6 (0.13)	26.5 (0.09)
Chemotherapy-related hospital admission, percentage points^c^	−1.15 (−1.42 to −0.88)	16.2 (0.11)	17.3 (0.08)
Avoidable ED visits, percentage points^c^	0.67 (0.43 to 0.92)	12.9 (0.10)	12.2 (0.07)
Preventable hospitalizations, percentage points^c^	−0.11 (−0.28 to 0.06)	5.8 (0.07)	5.9 (0.05)
Survival days	1.17 (−0.25 to 2.58)	432 (0.59)	431 (0.38)

Abbreviations: ED, emergency department; MA, Medicare Advantage; TM, traditional Medicare.

^a^ Weighted linear regression with county fixed effects.

^b^ This is a part of outpatient care.

^c^ Estimates were multiplied by 100 to show estimates as percentage point differences.

The difference in resource use for outpatient care was associated with different resource use for Part B chemotherapy: MA enrollees had $4765 (95% CI, $4547-$4982) lower Part B chemotherapy resource use compared with TM beneficiaries ($18 893 vs $23 658). Risk-adjusted resource use for Part B chemotherapy supportive drugs was $415 (95% CI, $396-$435) lower in MA than in TM ($2222 vs $2637). MA enrollees had higher resource use for Part D drugs than TM, but only by $403 (95% CI, $235-$572; $12 842 vs $12 439). This difference was largely due to a $337 (95% CI, $175-$498) higher resource use for Part D chemotherapy ($10 605 in MA vs $10 269 in TM).

Next, we examined the sources of the difference in resource use for Part B chemotherapy. MA enrollees had 1.03 (95% CI, 0.99-1.06) fewer chemotherapy visits than TM beneficiaries (5.92 vs 6.94). We multiplied the difference in visits by the average resource use per visit ($3377) in MA to estimate that fewer visits in MA resulted in $3478 lower resource use for part B chemotherapy. This is approximately 72% ($3478 of $4765) of the difference in resource use. MA enrollees received $227 (95% CI, $183-$272) less expensive chemotherapy per visit than TM enrollees ($3377 vs $3604). We multiplied the difference in resource use per visit by the average number of visits (5.92) in MA to estimate that less-expensive chemotherapy contributed $1345 to the lower resource use, approximately 28%.

### MA Enrollment and Quality

The association between MA enrollment and care quality was mixed (Table 2). MA enrollees had 2.9 percentage points (95% CI, 2.6-3.2 percentage points) less chemotherapy-related ED use than TM beneficiaries (23.6% vs 26.5%). This corresponds to 11% lower chemotherapy-related ED use in MA than the mean value. MA enrollees also had fewer chemotherapy-related hospitalizations: 1.2 percentage points (95% CI, 0.9-1.4 percentage points) (16.2% in MA vs 17.3% in TM) or 6.8% lower than the mean value. However, MA beneficiaries had 0.7 percentage point higher (12.9% in MA vs 12.2% in TM) avoidable ED use than TM beneficiaries (5.4% higher than mean), and MA had similar preventable hospitalizations to TM. Importantly, we found no significant difference in survival during 18 months after chemotherapy initiation.

### Results From Additional Analyses

**Figure 1.**
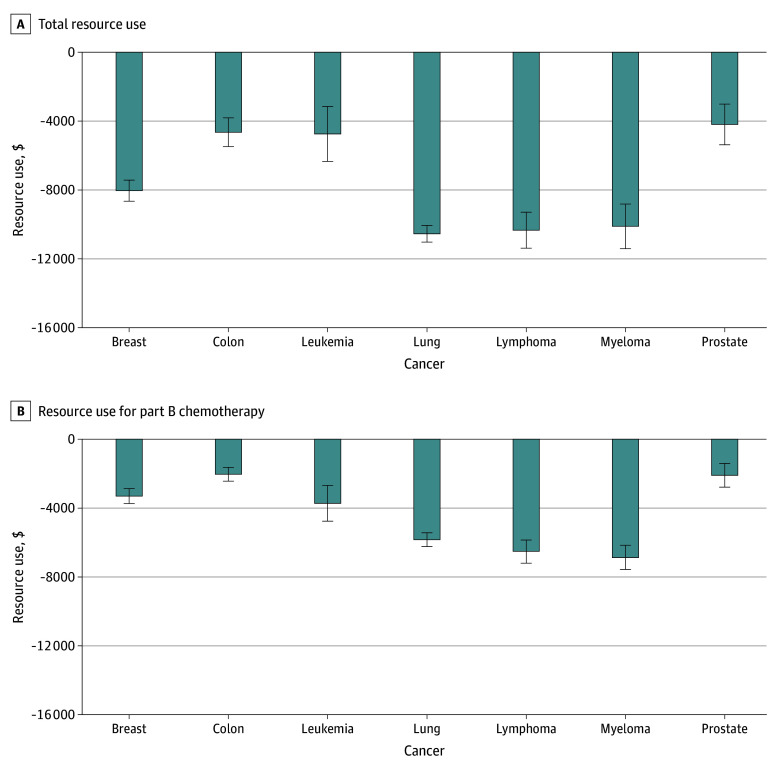
Difference in Total Resource Use and Resource Use for Part B Chemotherapy for Medicare Advantage vs Traditional Medicare by Cancer Type

The subgroup analyses by cancer type showed similar results to the main analyses (eTable 2 in Supplement 1). Figure 1 shows that MA enrollees had lower adjusted total resource use during the 6-month chemotherapy episode than TM beneficiaries across all 7 cancers. Reduction in total resource use in MA ranged from −$4194 for prostate cancer to −$10 545 for lung cancer. Across cancer types, this reduction was mainly associated with less resource use for Part B chemotherapy, which ranged from −$2030 for colon cancer to −$6865 for myeloma. Both fewer Part B chemotherapy visits and use of lower-cost chemotherapy contributed to the reduction in resource use for Part B chemotherapy in MA (eTable 3 in Supplement 1).

**Figure 2.**
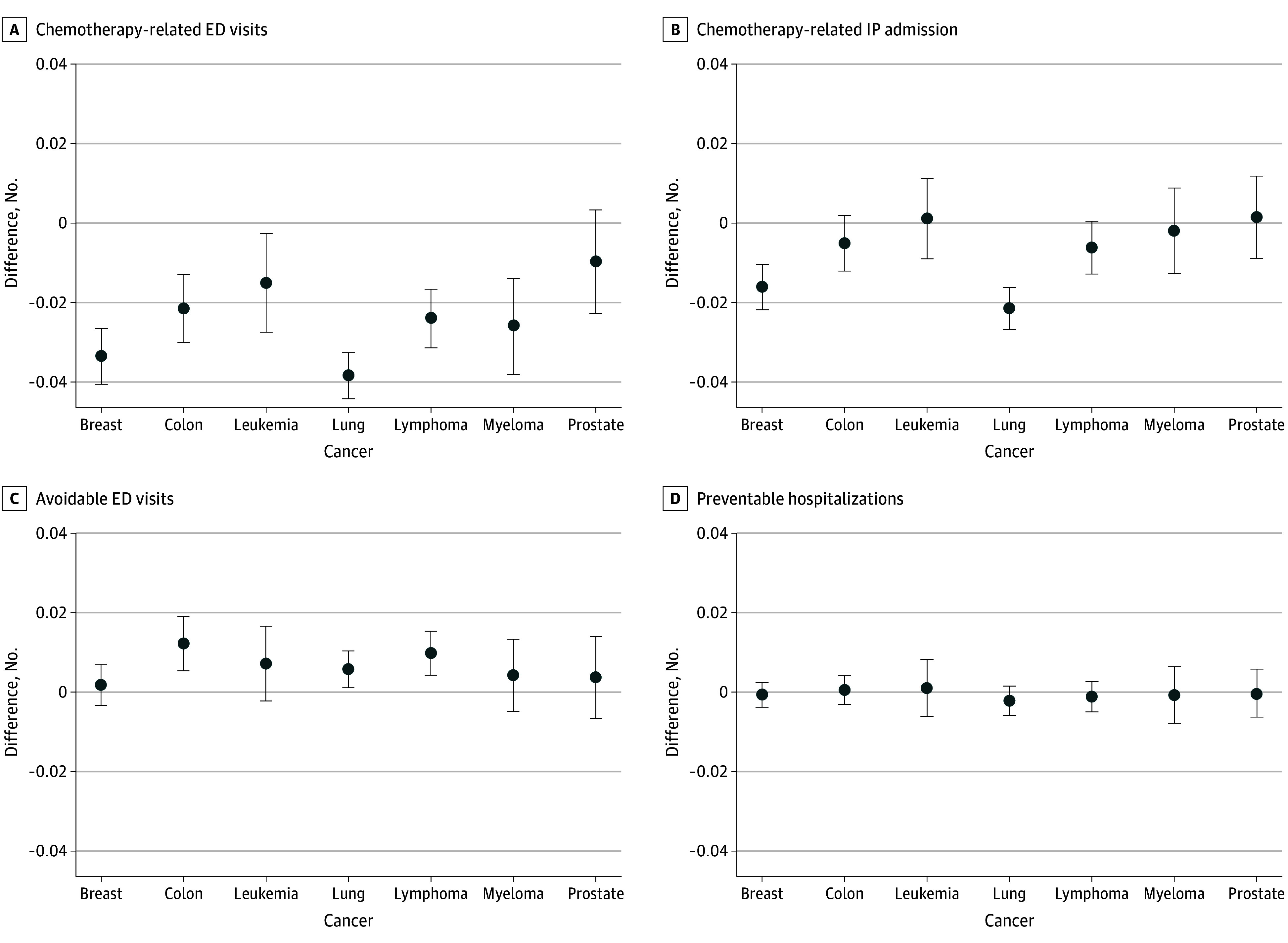
Differences in Chemotherapy-Related Quality and Adverse Health Events Between Medicare Advantage and Traditional Medicare by Cancer Type ED indicates emergency department; IP, inpatient.

**Figure 3.**
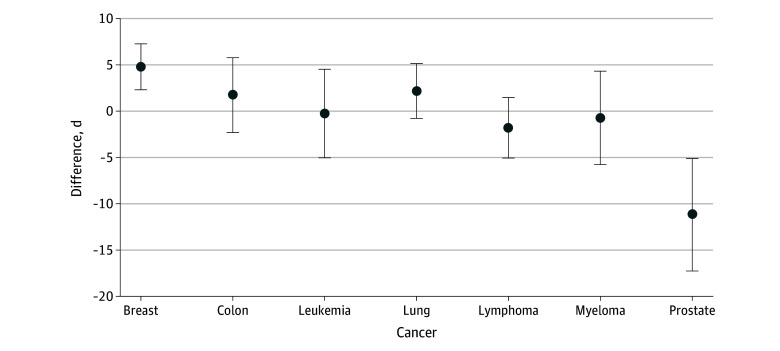
Differences in Survival Days Between Medicare Advantage and Traditional Medicare by Cancer Type

Like the overall analysis, the results on care quality by cancer type were mixed (regression coefficients are reported in eTable 2 in Supplement 1). Figure 2 shows that MA enrollees had fewer chemotherapy-related ED visits than TM beneficiaries for all cancer types (ranging from −3.84 percentage points for lung cancer to −1.50 percentage points for leukemia) except prostate cancer, where the results were not significant. There were no differences in chemotherapy-related hospitalizations or adverse health events from TM, for most cancer types. Figure 3 indicates that MA enrollees had 4.8-day longer survival (95% CI, 2.3 to 7.3 days) than TM beneficiaries (493 vs 488) for breast cancer but 11.2-day shorter survival (95% CI, −17.3 to −5.1 days) for prostate cancer (458 vs 469). While statistically significant, these small differences are not considered clinically meaningful.^27^

Findings from the subgroup analysis by MA plan type and sensitivity checks were consistent with the main analysis. Results of the additional analyses are provided in eTables 3 to 6 in Supplement 1.

## Discussion

MA patients receiving chemotherapy had significantly lower total resource use during the 6-month episode than their counterparts in TM. Lower resource use was observed for hospital inpatient services and outpatient care, with a substantial reduction in resource use for Part B chemotherapy. Lower resource use for Part B chemotherapy in MA resulted from both use of lower-cost chemotherapy drugs and fewer chemotherapy visits.

These findings are consistent with prior studies of the overall MA population or patients with diabetes.^3,4,5,6^ Our new finding is that MA plans had lower resource use than TM among enrollees with cancer undergoing chemotherapy—a serious condition managed by specialists and requiring expensive treatments. This suggests that MA’s cost advantages over TM are not limited to conditions for which low-cost primary care management can avoid costly services.^10^

A reduction in resource use in MA, even for patients with cancer receiving chemotherapy, is an expected outcome if MA plans apply managed care practices, such as care coordination and quantity limits to all patients. It could harm patient outcomes if MA limited necessary treatments for patients receiving chemotherapy. However, we found that MA enrollees did better on the 2 quality measures related to chemotherapy, implying that different patterns of chemotherapy use in MA did not affect chemotherapy-related quality. MA plans may have reduced clinically unnecessary services and used low-cost alternative chemotherapy regimens that do not harm patient outcomes. For example, a recent study^29^ found that MA enrollees generally used more biosimilars than TM beneficiaries. We also found that MA enrollees used less costly chemotherapy per visit than TM beneficiaries.

MA enrollees had slightly higher avoidable ED use than TM beneficiaries. However, they had similar rates of preventable hospitalizations and importantly, there was no difference in survival days through 18 months despite having lower resource use than TM. These findings are promising because they suggest that reducing resource use does not sacrifice survival.

It is also encouraging that our findings were consistent across 7 cancer types, MA plan types, and different specifications, given that MA has been steadily growing and now covers more than half of the Medicare population. They could be informative to CMS, which has increasingly promoted efficient delivery of cancer care through chemotherapy-episode based initiatives, such as the oncology care model or Enhancing Oncology Model.^20,21^ Learning about effective approaches used by MA plans could help CMS refine those initiatives to improve patient care while reducing costs, thereby increasing value for all Medicare beneficiaries.

While our findings are generally positive for MA, they do not tell us whether chemotherapy initiation differs between MA and TM. MA plans may apply strict prior authorization requirements for chemotherapy initiation and thereby limit the initiation of necessary treatments among patients with cancer. Our study did not examine this possibility; consequently, the results should be interpreted only for patients with cancer initiating chemotherapy. Future research into mechanisms by which MA influences treatment patterns for patients with cancer would be critical.

### Limitations

Our study has several limitations. First, we applied inverse-probability weighting to mitigate selection issues; however, the effectiveness of this approach is limited to balancing only observed characteristics. The findings could be affected by unobserved patient characteristics. Second, our study excludes MA enrollees in contracts with incomplete encounter data. Our results may not apply to the entire MA population. Third, our study does not apply to beneficiaries without Part D drug coverage. Fourth, we did not examine specific strategies and utilization management techniques that MA plans use to reduce resource use or improve quality during the chemotherapy episode. Fifth, our measures of quality are limited to those measured from the claims and encounter data, which lack patients’ cancer stage. We did not measure whether the care provided to the patient followed recommended guidelines. Furthermore, our measure of survival during an 18-month period following chemotherapy initiation might not adequately capture survival differences associated with adjuvant treatment of early-stage cancers.

## Conclusions

Using national Medicare data, we found that MA is associated with lowered resource use without reduced survival for patients with cancer receiving chemotherapy. As MA grows, continued efforts are needed to identify mechanisms by which MA affects treatment patterns for patients with cancer to develop targeted interventions that enhance patient care and resource efficiency in Medicare.

## Notes

Supplement 1. **eMethods.** Detailed Methodological Considerations **eTable 1.** Results From the Regression Analysis of Total Resource Use **eTable 2.** Regression Results and Adjusted Outcomes by Cancer Type **eTable 3.** Regression Results and Adjusted Outcomes by MA Plan Type **eTable 4.** Regression Results and Adjusted Outcomes: Using 12-Month Chemotherapy Episode **eTable 5.** Regression Results and Adjusted Outcomes from Analysis of Using First Chemotherapy Episode **eTable 6.** GLM for Resource Use and Logit for Quality Outcomes **eTable 7.** Drug List

Supplement 2.
Data Sharing Statement
