# Error in Data and Analyses

**DOI:** 10.1001/jamanetworkopen.2025.1566

**Published:** 2025-02-18

In the Original Investigation “Resource Use and Care Quality Differences Among Medicare Beneficiaries Undergoing Chemotherapy,”^1^ published September 20, 2024, there were errors resulting from the inadvertent removal of some patients who had died from the analysis. This error affected the number of participants and their demographic characteristics and the regression estimates for total resource use for Medicare Advantage and traditional Medicare beneficiaries as reported in the Results, Tables, Figures, and Supplement 1. However, the findings remain consistent with the originally published article. The article has been corrected^1^ and the authors have explained what occurred in a Comment.^2^
