# Supplementary material for: Resource Use and Care Quality Differences Among Medicare Beneficiaries Undergoing Chemotherapy
Source: JAMA Netw Open. 2024 Sep 20;7(9):e2434707. doi: 10.1001/jamanetworkopen.2024.34707 (PMC11415781; doi:10.1001/jamanetworkopen.2024.34707)
Supplement: Supplement 1. — eMethods. Detailed Methodological Considerations eTable 1. Results From the Regression Analysis of Total Resource Use eTable 2. Regression Results and Adjusted Outcomes by Cancer Type eTable 3. Regression Results and Adjusted Outcomes by MA Plan Type eTable 4. Regression Results and Adjusted Outcomes: Using 12-Month Chemotherapy Episode eTable 5. Regression Results and Adjusted Outcomes from Analysis of Using First Chemotherapy Episode eTable 6. GLM for Resource Use and Logit for Quality Outcomes eTable 7. Drug List [file jamanetwopen-e2434707-s001.pdf]

Supplemental Online Content

Kalidindi Y, Jung J, Feldman R, Carlin C, Song G, Mitchell A. Resource use and care quality differences among Medicare beneficiaries undergoing chemotherapy. *JAMA Netw Open.* 2024;7(9):e2434707. doi:10.1001/jamanetworkopen.2024.34707

eMethods. Detailed Methodological Considerations

eTable 1. Results From the Regression Analysis of Total Resource Use

eTable 2. Regression Results and Adjusted Outcomes by Cancer Type

eTable 3. Regression Results and Adjusted Outcomes by MA Plan Type

eTable 4. Regression Results and Adjusted Outcomes: Using 12-Month Chemotherapy Episode

eTable 5. Regression Results and Adjusted Outcomes from Analysis of Using First Chemotherapy Episode

eTable 6. GLM for Resource Use and Logit for Quality Outcomes

eTable 7. Drug List

This supplemental material has been provided by the authors to give readers additional information about their work.

© 2024 Kalidindi Y et al. *JAMA Network Open*.

eMethods. Detailed Methodological Considerations

Sample and Episode Definitions

Our sample comprised Medicare beneficiaries who had one of seven cancer types: breast cancer, chronic leukemia, colorectal or small intestine cancer, lung cancer, lymphoma, multiple myeloma, and prostate cancer, and who initiated chemotherapy for the first time between January 1, 2016, and July 31, 2019. Beneficiaries must meet the requirement of at least a 1-year chemotherapy washout period, and must be observed for at least 1-year after chemotherapy was initiated, or until death if earlier. Chemotherapy included cytotoxic, targeted and immuno-therapy, and patients who were only on hormonal therapy were excluded.

A beneficiary could have two types of cancer, and we assigned cancer type associated with the initial chemotherapy. Specifically, we followed an approach similar to Oncology Care Model (OCM), an initiative by the Centers for Medicare and Medicaid (CMS).[1] Note that while the original OCM evaluated 24 cancer types, the more recent Enhancing Oncology Model (EOM)[2] focuses on seven cancer types: breast cancer, chronic leukemia, colorectal and small intestine cancer, lung cancer, lymphoma, multiple myeloma, and prostate cancer. Thus, we selected the study sample with any of the seven cancer types.

Chemotherapy Initiation:

We examined both Part B (Outpatient, Carrier, and Durable Medical Equipment, Prosthetics/Orthotics, and Supplies [DMEPOS]) and Part D claims files for a chemotherapy initiation claim:

1) For a Part B claim to qualify as an initiation event – we required the claim to have both an initiating cancer therapy (listed in OCM initiating therapies list document) and a cancer diagnosis for one of the seven cancer types (listed in the OCM cancer type

© 2024 Kalidindi Y et al. *JAMA Network Open*.

mapping and codes document). We also required that the Part B claim does not have a place of service (POS) code='21', which indicates an inpatient hospital setting.

2) For a Part D claim to qualify as an initiation event – we required a Part D claim with an initiating cancer therapy (listed in OCM initiating therapies list document) and a Part B claim with an included cancer diagnosis (listed in the OCM cancer type mapping and codes document) on the prescription fill date or in the 59 days preceding the fill date with a POS code not indicating inpatient hospital.

Sample Selection:

We applied sample selection in two steps – 1) the first step required that the beneficiary meets criterion outlined below for a 6-month chemotherapy-free period before the initiation event (also referred to as the wash-out period), and a 6-month follow-up period following chemotherapy initiation, and 2) the second step involved extending the wash-out and follow-up periods to 1-year. Our decision to extend the washout and follow-up period to 1-year was to ensure that the results from our main analysis and sensitivity analysis were comparable. The sample section criterion is outlined below:

- Beneficiary has continuous Part A/B/D coverage.
- Beneficiary is enrolled exclusively in either Medicare Advantage (MA) or Traditional Medicare (TM).
- Beneficiary does not receive the Medicare End Stage Renal Disease (ESRD) benefit.
- Beneficiary has at least one qualifying Evaluation & Management (E&M) visit. A qualifying E&M visit is defined as having a HCPCS code in the ranges 99201-99205 or 99211-99215, a cancer diagnosis for one of the seven cancer types included in the document "OCM Cancer Type Mapping and Codes," and billed by a TIN with at least

© 2024 Kalidindi Y et al. *JAMA Network Open*.

one oncology provider in the performance period. Oncology providers are those with a specialty code of Hematology/Oncology, Medical Oncology, Surgical Oncology, Radiation Oncology, and/or Gynecological/Oncology.

Note on switching:

A small number of observation (1.6% or 9,910 observations) were dropped due to switching when we extended our wash-out and follow-up periods from 6-month period to 1-year.

- 26.5% (2,623 of the 9,910 switchers) moved from TM to MA between 6 months and 12 months prior to chemotherapy initiation (i.e., during the extended pre-chemotherapy wash-out period).
- 14.8% (1,469 of the 9,910 switchers) moved from MA to TM during the extended pre-chemotherapy wash-out period.
- 30.5% (3,024 of the 9,910 switchers) moved from MA to TM between 6 months and 12 months after the chemotherapy initiation (i.e. during the extended post-chemotherapy period).
- 28.2% (2,794 out of the 9,910 switchers) moved from MA to TM during the post-chemotherapy period. Including these switchers in the analysis did not change the results.

© 2024 Kalidindi Y et al. *JAMA Network Open*.

The steps in sample selection are shown below:

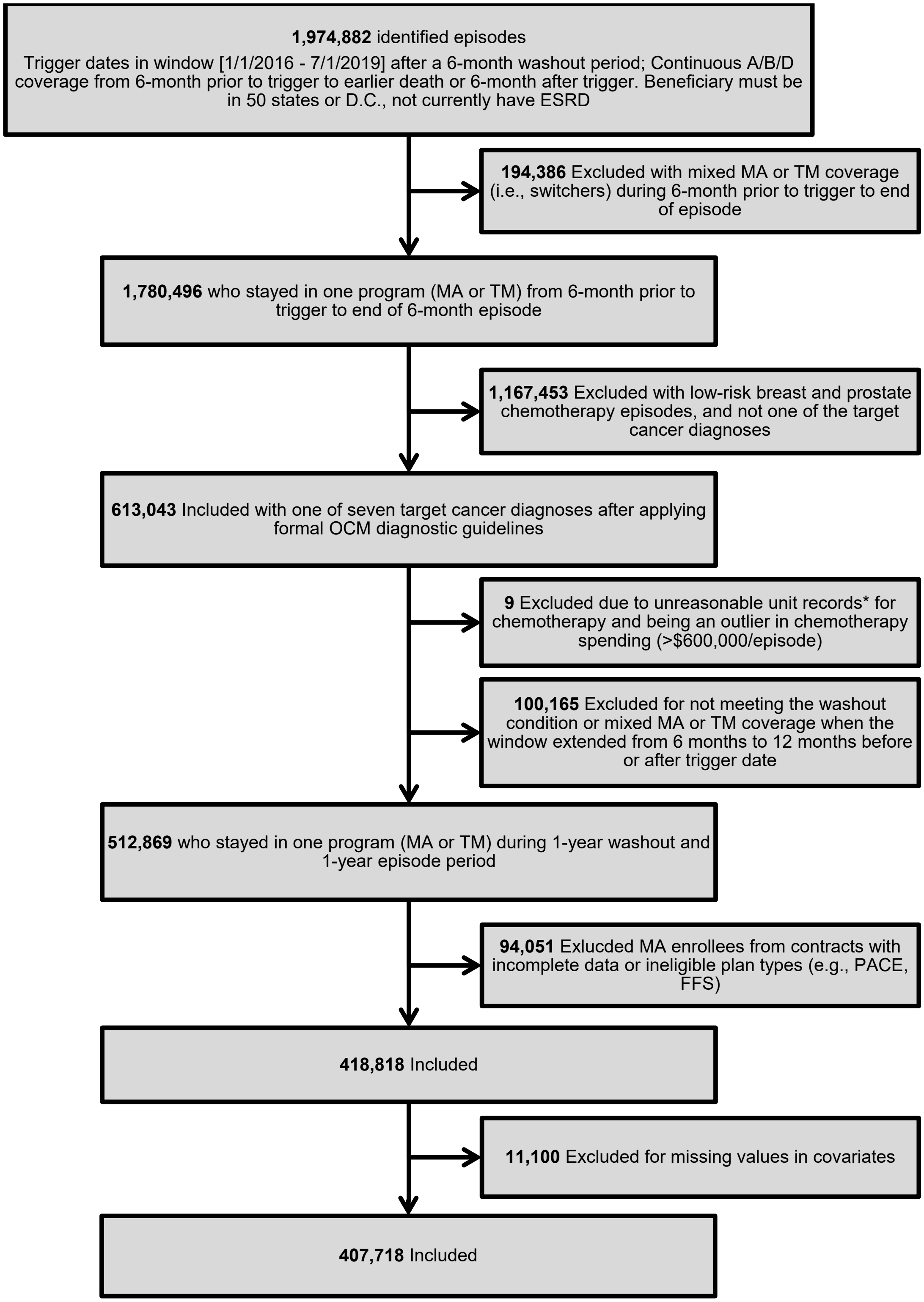

© 2024 Kalidindi Y et al. *JAMA Network Open*.

Cancer Type Assignment:

Following the OCM approach, we assigned cancer type using the plurality of diagnosis on qualifying E&M visits in the carrier file that occurred during the 6-month episode. The diagnosis codes mapping by cancer type is listed in the OCM Cancer Type Mapping and Codes.

Quality Outcomes

Chemotherapy-related outcomes:

We adapted the CMS measure of chemotherapy-associated hospitalizations and ED visits.[3] These measures identify hospital admissions and ED visits for 10 potentially preventable conditions within 30 days of receiving outpatient chemotherapy. The ten conditions are anemia, nausea, vomiting, dehydration, neutropenia, diarrhea, pain, pneumonia, fever, and sepsis. While the measure was originally developed for patients receiving chemotherapy in hospital outpatient settings, we expanded the measure to also include chemotherapy delivered in physicians' offices or for patients receiving oral chemotherapy covered under Part D similar to Keating et al (2021).[4]

Adverse Health Events:

We created variables for avoidable Emergency Department (ED) visits and preventable hospitalizations. We identify avoidable ED visits using the algorithm developed by Billings,[5] with updated diagnosis code mappings developed by Johnson, et al (2017).[6] The Billings algorithm identifies claims related to injury, alcohol, drugs, psych, and then of the remainder it assigns probabilities of the ED visit to one of four categories: 1) non-emergent; 2) emergent but primary care treatable; 3) emergent and ED needed, but preventable or avoidable; and 4) emergent and ED needed, not preventable or avoidable. We defined avoidable ED visit as the probability of being in the fourth category (emergent, not avoidable) being equal to zero, to help identify high-acuity visits based on "clinical" needs.

© 2024 Kalidindi Y et al. *JAMA Network Open*.

To identify preventable hospitalizations, we use the Prevention Quality Indicator (PQI) algorithm developed by the Agency for Healthcare Research and Quality.[7] This algorithm constructs an indicator of admissions due to conditions that are potentially preventable when a patient has access to high-quality ambulatory care. Specifically, the PQI algorithm identifies hospital admissions for one of the following conditions: diabetes with short-term complications, diabetes with long-term complications, uncontrolled diabetes without complications, diabetes with lower-extremity amputation, chronic obstructive pulmonary disease, asthma, hypertension, heart failure, bacterial pneumonia, or urinary tract infection.

Survival:

To measure survival, we obtained the mean survival time in days from the chemotherapy initiation date through 18 months. For this measure, we required continuous TM or continuous MA coverage through the end of the observation period, up to 18 months after chemotherapy initiation.

Explanatory Variables

Below we list explanatory variables and the datasets used to construct those covariates

1) Demographics: age (age groups: <65, 65-69,70-74,75-79,80-84,>=85), gender, race (non-Hispanic White, non-Hispanic Black, Hispanic, Asian, and other race), and Medicare-Medicaid dual-eligibility. These were obtained from the Medicare Beneficiary Summary File (MBSF)
2) Health Risk measures: Cancer metastasis, claims-based frailty index, and a summary health-risk score measured by hierarchical condition category (HCC) scores. To construct the general cancer metastatic status, we looked for ICD codes indicating metastasis (C77-C79) during the wash-out period including the trigger date to measure the baseline

© 2024 Kalidindi Y et al. *JAMA Network Open*.

characteristics. We required patients to have at least 2 claims of metastatic status in OP or Carrier. Second, claims-based frailty index was constructed following Kim et al. (2018).[8] This index was used to account for functional limitations and differences in the propensity to receive chemotherapy treatments. Finally, we used Hierarchical Condition Category (HCC) scores. This summary risk measure is used by CMS to adjust MA payments. We computed HCC scores using the v22 CMS-HCC software, excluding Health Risk Assessment (HRA)/chart review records for MA enrollees and HRA records for TM beneficiaries.

3) ZIP-level rurality of residence based on Rural-Urban Commuting Area codes.
4) County-level health care provider-related variables were obtained from Area Health Resources File (AHRF), including hospital beds per 1000 residents, physicians per 1000, and skilled nursing facility beds per 1000.
5) Zip-level socioeconomic variables were supplied from the American Community Survey (ACS) data, including percentage of adults with a 4-year college degree, percentage of households living under the Federal Poverty Limit, and the percentage age 5 or older speaking English only.

Statistical Analysis

Inverse Probability Treatment Weighting:

We used a logistic regression model to estimate the propensity for enrolling in MA based on all the explanatory variables listed in the previous section. From the logit model, we obtained the inverse probability of treatment weights (IPTW). We then estimated the linear regression using IPTW as the weight. The regression model included an indicator of MA enrollment, patient and residence (ZIP or county) factors, and county fixed effects. Standard errors were clustered within

© 2024 Kalidindi Y et al. *JAMA Network Open*.

counties.

Calculating Adjusted Outcomes:

Using the regression results, we calculated the risk-adjusted outcome values in each group. To calculate risk-adjusted outcomes in MA, we computed predicted outcomes by setting the MA indicator to one and all other covariates to their mean values. Similarly, we computed the outcomes in TM by calculating the predicted outcomes with the MA indicator equal to zero and the means of all other covariates.

References:

1. Centers for Medicare & Medicaid Services. Oncology Care Model Performance-Based Payment Methodology Version 7.1. Centers for Medicare & Medicaid Services (CMS); 2021. Accessed April 12, 2024.

2. Centers for Medicare & Medicaid Services. Enhancing Oncology Model. Centers for Medicare & Medicaid Services (CMS). Published 2023. Accessed April 12, 2024. https://www.cms.gov/priorities/innovation/innovation-models/enhancing-oncology-model

3. Centers for Medicare & Medicaid Services. Hospital Outpatient Quality Reporting Program. Centers for Medicare & Medicaid Services (CMS). Accessed April 12, 2024. https://www.cms.gov/medicare/quality/initiatives/hospital-quality-initiative/hospital-outpatient-quality-reporting-program

4. Keating NL, Jhatakia S, Brooks GA, et al. Association of Participation in the Oncology Care Model With Medicare Payments, Utilization, Care Delivery, and Quality Outcomes. *JAMA*. 2021;326(18):1829-1839. doi:10.1001/jama.2021.17642

5. Billings J. ED Utilization Background. NYU Wagner. Published 2013. https://wagner.nyu.edu/faculty/billings/nyued-articles

© 2024 Kalidindi Y et al. *JAMA Network Open*.

6. Johnston KJ, Allen L, Melanson TA, Pitts SR. A "Patch" to the NYU Emergency Department Visit Algorithm. *Health Services Research*. 2017;52(4):1264-1276. doi:10.1111/1475-6773.12638

7. Agency for Healthcare Research and Quality. AHRQ QI: Prevention Quality Indicators Overview. Accessed April 12, 2024. https://qualityindicators.ahrq.gov/measures/pqi_resources

8. Kim DH, Schneeweiss S, Glynn RJ, Lipsitz LA, Rockwood K, Avorn J. Measuring Frailty in Medicare Data: Development and Validation of a Claims-Based Frailty Index. *J Gerontol A Biol Sci Med Sci*. 2018;73(7):980-987. doi:10.1093/gerona/glx229

© 2024 Kalidindi Y et al. *JAMA Network Open*.

eTable 1. Results From the Regression Analysis of Total Resource Use

	Regression results[a]
	Coefficient (95% CI)
Variables, US$	
MA	-8,498 (-8,817 to -8,178)
Female	-3,870 (-4,221 to -3,519)
Age, y	
< 64	Ref
65 to < 70	-2,121 (-2,731 to -1,511)
70 to < 75	-4,228 (-4,824 to -3,633)
75 to < 80	-7,331 (-7,993 to -6,668)
80 to < 85	-11,772 (-12,461 to -11,082)
≥ 85	-19,096 (-19,853 to -18,339)
Race	
Asian or Pacific Islander	219 (-2,122 to 2,560)
Hispanic	-1,214 (-1,913 to -515)
Non-Hispanic Black	209 (-322 to 739)
Non-Hispanic White	Ref
Other[b]	-1,407 (-2,966 to 151)
Cancer type	
Breast	17,197 (16,692 to 17,702)
Colorectal	-8,973 (-9,682 to -8,265)
Leukemia	32,631 (31,830 to 33,431)
Lung	19,473 (19,002 to 19,944)
Lymphoma	37,935 (37,365 to 38,506)
Myeloma	60,386 (59,656 to 61,116)
Prostate	Ref
Dual eligible	597 (120 to 1,075)
Metastatic cancer	4,679 (4,328 to 5,030)
Frailty index	-2,285 (-5,011 to 442)
HCC score	
0 to < 1	Ref
1 to < 2	2,668 (2,065 to 3,272)
2 to < 3	5,652 (5,051 to 6,253)
≥ 4	8,615 (7,910 to 9,320)
County-level health care resources)	
Beds/1000, No.	50 (-108 to 208)
Doctors/1000, No.	77 (-155 to 309)
SNF Beds/1000, No.	13 (-140 to 166)
Rural	-263 (-1,066 to 541)
ZIP code demographics	
College educated	55 (37 to 73)
Speaking English only	-4 (-20 to 12)
Under federal poverty level	4 (-26 to 35)

Abbreviations: MA, Medicare Advantage; HCC, Hierarchical Condition Category; SNF, Skilled nursing facility; CI, confidence interval.

[a] Weighted linear regressions with county fixed effects.

[b] Other race is a category defined in the Master Beneficiary Summary File (MBSF).

© 2024 Kalidindi Y et al. *JAMA Network Open*.

eTable 2. Regression Results and Adjusted Outcomes by Cancer Type

Outcome	Breast Cancer: Regression results, coefficient (95% CI)[a]	Breast Cancer: Adjusted means (SE) MA	Breast Cancer: Adjusted means (SE) TM	Colorectal Cancer: Regression results, coefficient (95% CI)[a]	Colorectal Cancer: Adjusted means (SE) MA	Colorectal Cancer: Adjusted means (SE) TM
Resource use, US $						
Total resource use	-8,043 (-8,657 to -7,429)	53,147 (237.38)	61,190 (176.02)	-4,643 (-5,477 to -3,810)	41,618 (381.38)	46,261 (183.87)
By service type						
Hospital inpatient services	-594 (-811 to -376)	5,282 (83.05)	5,875 (64.89)	-908 (-1,257 to -558)	9,621 (137.73)	10,529 (99.21)
Outpatient care	-8,224 (-8,769 to -7,678)	37,248 (208.14)	45,472 (159.08)	-4,350 (-5,066 to -3,634)	28,175 (340.69)	32,525 (142.27)
Prescription drugs	760 (386 to 1,134)	10,366 (148.59)	9,607 (105.07)	564 (342 to 787)	3,215 (89.74)	2,650 (54.90)
Hospice services	15 (-16 to 46)	251 (12.57)	236 (8.24)	50 (-12 to 112)	607 (26.10)	557 (14.90)
Chemotherapy services*						
Chemotherapy services, US $						
Part B chemotherapy	-3,295 (-3,728 to -2,861)	14,333 (164.44)	17,627 (131.71)	-2,030 (-2,431 to -1,630)	8,762 (157.40)	10,792 (101.96)
Part B chemotherapy- supportive drugs	-569 (-608 to -529)	2,173 (15.14)	2,742 (11.79)	-369 (-441 to -297)	3,531 (28.54)	3,900 (20.26)
Part D chemotherapy	529 (167 to 890)	8,169 (143.56)	7,640 (101.76)	472 (298 to 645)	1,226 (70.43)	755 (40.64)
Sources of differences in part B chemotherapy						
Chemotherapy visits per episode	-1.25 (-1.33 to -1.16)	5.86 (0.03)	7.11 (0.03)	-0.94 (-1.05 to -0.84)	7.10 (0.04)	8.04 (0.03)
Resource use/chemotherapy visit, US $	-91 (-152 to -29)	2,210 (24.96)	2,300 (16.90)	-24 (-87 to 39)	1,343 (25.22)	1,367 (14.59)
Quality of care						
Chemotherapy-related ED visits, percentage points[b]	-3.35 (-4.05 to -2.65)	20.79 (0.27)	24.14 (0.21)	-2.15 (-3.00 to -1.29)	21.93 (0.34)	24.08 (0.24)
Chemotherapy-related hospital admission, percentage points[b]	-1.61 (-2.18 to -1.04)	12.04 (0.22)	13.65 (0.17)	-0.51 (-1.21 to 0.19)	13.16 (0.28)	13.66 (0.19)
Avoidable ED visits, percentage points[b]	0.18 (-0.33 to 0.70)	10.12 (0.21)	9.93 (0.15)	1.22 (0.54 to 1.90)	13.16 (0.28)	11.94 (0.18)
Preventable hospitalizations, percentage points[b]	-0.07 (-0.38 to 0.24)	3.37 (0.12)	3.44 (0.09)	0.05 (-0.31 to 0.41)	3.09 (0.15)	3.04 (0.10)
Survival days	4.78 (2.30 to 7.27)	493 (0.96)	488 (0.72)	1.73 (-2.30 to 5.77)	441 (1.60)	440 (1.08)

*This is a part of outpatient care

Abbreviations: MA, Medicare Advantage; TM, Traditional Medicare; CI, confidence interval; SE, standard error.

[a] Weighted linear regressions with county fixed effects.

[b] Estimates were multiplied by 100 to show estimates as percentage point differences.

© 2024 Kalidindi Y et al. *JAMA Network Open.*

eTable 2 (continued). Regression Results and Adjusted Outcomes by Cancer Type

Outcome	Leukemia: Regression results, coefficient (95% CI)[a]	Leukemia: Adjusted means (SE) MA	Leukemia: Adjusted means (SE) TM	Lung Cancer: Regression results, coefficient (95% CI)[a]	Lung Cancer: Adjusted means (SE) MA	Lung Cancer: Adjusted means (SE) TM
Resource use, US $						
Total resource use	-4,744 (-6,341 to -3,148)	68,986 (662.80)	73,730 (350.64)	-10,545 (-11,029 to -10,062)	58,661 (190.19)	69,207 (143.13)
By service type						
Hospital inpatient services	-413 (-913 to 87)	7,082 (191.37)	7,495 (142.15)	-771 (-993 to -549)	10,639 (87.77)	11,410 (64.96)
Outpatient care	-5,785 (-7,235 to -4,336)	26,312 (602.86)	32,098 (319.20)	-9,800 (-10,227 to -9,373)	41,186 (164.68)	50,986 (130.49)
Prescription drugs	1,406 (354 to 2,458)	35,383 (411.78)	33,977 (287.67)	-67 (-286 to 152)	5,832 (87.31)	5,899 (61.50)
Hospice services	48 (-1 to 97)	209 (20.64)	161 (11.15)	92 (51 to 134)	1,004 (17.02)	912 (11.20)
Chemotherapy services*						
Chemotherapy services, US $						
Part B chemotherapy	-3,716 (4,755 to -2,677)	15,995 (455.25)	19,711 (257.70)	-5,828 (-6,227 to -5,429)	20,480 (153.19)	26,308 (122.99)
Part B chemotherapy- supportive drugs	-248 (-290 to -206)	794 (15.59)	1,042 (12.62)	-482 (-508 to -455)	2,282 (10.47)	2,764 (7.76)
Part D chemotherapy	1,472 (435 to 2,510)	33,396 (407.40)	31,924 (282.63)	-89 (-293 to 115)	3,509 (80.70)	3,598 (57.83)
Sources of differences in part B chemotherapy						
Chemotherapy visits per episode	-0.58 (-0.71 to -0.45)	2.73 (0.05)	3.31 (0.04)	-0.92 (-0.98 to -0.86)	5.96 (0.02)	6.88 (0.02)
Resource use/chemotherapy visit, US $	-173 (-394 to 49)	3,182 (97.63)	3,355 (43.68)	-409 (-483 to -334)	3,858 (31.25)	4,267 (18.43)
Quality of care						
Chemotherapy-related ED visits, percentage points[b]	-1.50 (-2.75 to -0.26)	20.34 (0.48)	21.85 (0.34)	-3.84 (-4.42 to -3.26)	26.71 (0.23)	30.55 (0.17)
Chemotherapy-related hospital admission, percentage points[b]	0.11 (-0.90 to 1.12)	12.41 (0.40)	12.30 (0.27)	-2.15 (-2.68 to -1.62)	20.02 (0.21)	22.16 (0.15)
Avoidable ED visits, percentage points[b]	0.72 (-0.22 to 1.66)	11.25 (0.37)	10.53 (0.26)	0.57 (0.11 to 1.04)	15.43 (0.19)	14.85 (0.13)
Preventable hospitalizations, percentage points[b]	0.10 (-0.61 to 0.82)	5.66 (0.28)	5.56 (0.19)	-0.22 (-0.59 to 0.15)	8.73 (0.15)	8.94 (0.10)
Survival days	-0.27 (-5.05 to 4.52)	483 (1.85)	483 (1.28)	2.17 (-0.80 to 5.14)	357 (1.21)	355 (0.82)

*This is a part of outpatient care

Abbreviations: MA, Medicare Advantage; TM, Traditional Medicare; CI, confidence interval; SE, standard error.

[a] Weighted linear regressions with county fixed effects.

[b] Estimates were multiplied by 100 to show estimates as percentage point differences.

© 2024 Kalidindi Y et al. *JAMA Network Open.*

eTable 2 (continued). Regression Results and Adjusted Outcomes by Cancer Type

Outcome	Lymphoma			Myeloma		
	Regression results, coefficient (95% CI)[a]	Adjusted means (SE)		Regression results, coefficient (95% CI)[a]	Adjusted means (SE)	
		MA	TM		MA	TM
Resource use, US $						
Total resource use	-10,336 (-11,382 to -9,289)	72,205 (423.78)	82,541 (225.10)	-10,115 (-11,417 to -8,814)	96,002 (496.19)	106,118 (384.23)
By service type						
Hospital inpatient services	-699 (-1,068 to -330)	10,464 (145.93)	11,163 (104.21)	-712 (-1,287 to -137)	12,837 (220.44)	13,549 (163.69)
Outpatient care	-9,656 (-10,612 to -8,701)	53,387 (386.57)	63,043 (196.92)	-9,905 (-10,732 to -9,079)	34,829 (299.28)	44,735 (262.17)
Prescription drugs	-8 (-413 to 397)	8,038 (159.91)	8,046 (109.33)	474 (-563 to 1,511)	47,912 (399.53)	47,438 (302.31)
Hospice services	27 (-7 to 61)	317 (14.48)	290 (8.57)	28 (-34 to 90)	424 (25.45)	396 (16.94)
Chemotherapy services*						
Chemotherapy services, US $						
Part B chemotherapy	-6,499 (-7,192 to -5,807)	32,523 (280.38)	39,022 (157.29)	-6,865 (-7,562 to -6,167)	20,613 (250.30)	27,478 (222.50)
Part B chemotherapy- supportive drugs	-421 (-485 to -357)	2,414 (26.05)	2,835 (13.12)	-394 (-439 to -350)	1,334 (16.43)	1,728 (13.50)
Part D chemotherapy	-137 (-512 to 238)	5,642 (148.49)	5,779 (100.16)	565 (-456 to 1,586)	45,401 (393.20)	44,836 (297.62)
Sources of differences in part B chemotherapy						
Chemotherapy visits per episode	-0.95 (-1.02 to -0.87)	5.28 (0.03)	6.22 (0.02)	-1.94 (-2.16 to -1.72)	8.93 (0.08)	10.87 (0.07)
Resource use/chemotherapy visit, US $	-163 (-349 to 23)	6,433 (74.75)	6,597 (30.09)	-207 (-297 to -117)	2,246 (35.76)	2,453 (22.93)
Quality of care						
Chemotherapy-related ED visits, percentage points[b]	-2.40 (-3.14 to -1.66)	21.92 (0.30)	24.32 (0.21)	-2.60 (-3.81 to -1.39)	29.58 (0.46)	32.18 (0.35)
Chemotherapy-related hospital admission, percentage points[b]	-0.62 (-1.28 to 0.05)	16.76 (0.27)	17.37 (0.18)	-0.19 (-1.27 to 0.88)	20.82 (0.42)	21.01 (0.31)
Avoidable ED visits, percentage points[b]	0.98 (0.43 to 1.53)	11.44 (0.23)	10.46 (0.15)	0.42 (-0.49 to 1.33)	14.37 (0.36)	13.95 (0.26)
Preventable hospitalizations, percentage points[b]	-0.12 (-0.50 to 0.26)	4.80 (0.16)	4.91 (0.10)	-0.07 (-0.79 to 0.64)	7.72 (0.28)	7.79 (0.20)
Survival days	-1.80 (-5.07 to 1.46)	462 (1.33)	463 (0.86)	-0.74 (-5.79 to 4.31)	458 (1.96)	459 (1.38)

*This is a part of outpatient care

Abbreviations: MA, Medicare Advantage; TM, Traditional Medicare; CI, confidence interval; SE, standard error.

[a] Weighted linear regressions with county fixed effects.

[b] Estimates were multiplied by 100 to show estimates as percentage point differences.

© 2024 Kalidindi Y et al. *JAMA Network Open.*

eTable 2 (continued). Regression Results and Adjusted Outcomes by Cancer Type

	Prostate Cancer		
		Adjusted means (SE)	
Outcome	Regression results, coefficient (95% CI)[a]	MA	TM
Resource use, US $			
Total resource use	-4,194 (-5,375 to -3,012)	50,045 (432.48)	54,238 (356.86)
By service type			
Hospital inpatient services	-481 (-981 to 20)	6,175 (185.70)	6,656 (146.22)
Outpatient care	-5,196 (-6,056 to -4,335)	20,742 (309.93)	25,937 (266.54)
Prescription drugs	1,440 (576 to 2,303)	22,845 (322.00)	21,405 (254.92)
Hospice services	43 (-16 to 102)	283 (21.95)	240 (16.24)
Chemotherapy services*			
Chemotherapy services, US $			
Part B chemotherapy	-2,088 (-2,779 to -1,397)	6,167 (246.90)	8,255 (222.97)
Part B chemotherapy- supportive drugs	-210 (-273 to -147)	840 (23.22)	1,050 (18.47)
Part D chemotherapy	1,539 (689 to 2,389)	21,011 (318.56)	19,472 (248.32)
Sources of differences in part B chemotherapy			
Chemotherapy visits per episode	-0.79 (-0.93 to -0.64)	3.94 (0.05)	4.73 (0.04)
Resource use/chemotherapy visit, US $	-215 (-370 to -59)	1,509 (58.05)	1,724 (48.52)
Quality of care			
Chemotherapy-related ED visits, percentage points[b]	-0.97 (-2.27 to 0.33)	18.61 (0.48)	19.59 (0.39)
Chemotherapy-related hospital admission, percentage points[b]	0.15 (-0.89 to 1.18)	10.68 (0.38)	10.53 (0.30)
Avoidable ED visits, percentage points[b]	0.37 (-0.66 to 1.40)	10.91 (0.38)	10.55 (0.30)
Preventable hospitalizations, percentage points[b]	-0.02 (-0.63 to 0.58)	3.58 (0.23)	3.60 (0.18)
Survival days	-11.20 (-17.28 to -5.13)	458 (2.27)	469 (1.67)

*This is a part of outpatient care

Abbreviations: MA, Medicare Advantage; TM, Traditional Medicare; CI, confidence interval; SE, standard error.

[a] Weighted linear regressions with county fixed effects.

[b] Estimates were multiplied by 100 to show estimates as percentage point differences.

© 2024 Kalidindi Y et al. *JAMA Network Open.*

eTable 3. Regression Results and Adjusted Outcomes by MA Plan Type

Outcome	HMO			PPO		
	Regression results, coefficient (95% CI)[a]	Adjusted means (SE)		Regression results, coefficient (95% CI)[a]	Adjusted means (SE)	
		MA	TM		MA	TM
Resource use, US $						
Total resource use	-8,909 (-9,429 to -8,389)	60,720 (218.35)	69,629 (100.70)	-7,883 (-8,273 to -7,492)	61,464 (172.32)	69,347 (84.74)
By service type						
Hospital inpatient services	-788 (-963 to -612)	9,204 (74.22)	9,992 (42.09)	-538 (-718 to -359)	9,391 (79.15)	9,930 (38.05)
Outpatient care	-7,652 (-8,121 to -7,182)	38,919 (196.45)	46,571 (87.32)	-8,640 (-8,951 to -8,330)	37,971 (135.30)	46,612 (71.74)
Prescription drugs	-552 (-777 to -327)	12,007 (95.64)	12,558 (53.53)	1,267 (1,022 to 1,512)	13,558 (108.69)	12,291 (49.52)
Hospice services	82 (56 to 108)	590 (11.55)	508 (5.75)	29 (0 to 57)	543 (13.20)	514 (5.14)
Chemotherapy services*						
Chemotherapy services, US $						
Part B chemotherapy	-4,327 (-4,665 to -3,988)	19,295 (142.93)	23,621 (71.14)	-5,157 (-5,423 to -4,890)	18,524 (115.57)	23,681 (62.32)
Part B chemotherapy- supportive drugs	-327 (-355 to -298)	2,307 (11.96)	2,634 (6.05)	-479 (-503 to -455)	2,156 (10.48)	2,635 (5.24)
Part D chemotherapy	-382 (-597 to -168)	9,941 (91.56)	10,324 (51.19)	972 (738 to 1,207)	11,134 (104.08)	10,161 (47.56)
Sources of differences in part B chemotherapy						
Chemotherapy visits per episode	-0.91 (-0.96 to -0.86)	6.03 (0.02)	6.94 (0.01)	-1.13 (-1.18 to -1.08)	5.80 (0.02)	6.92 (0.01)
Resource use/chemotherapy visit, US $	-157 (-244 to -70)	3,431 (35.70)	3,587 (13.13)	-281 (-326 to -237)	3,341 (20.03)	3,622 (9.60)
Quality of care						
Chemotherapy-related ED visits, percentage points[b]	-2.46 (-2.89 to -2.03)	24.15 (0.19)	26.61 (0.10)	-3.25 (-3.69 to -2.82)	23.15 (0.19)	26.40 (0.09)
Chemotherapy-related hospital admission, percentage points[b]	-1.11 (-1.48 to -0.74)	16.27 (0.16)	17.38 (0.09)	-1.10 (-1.48 to -0.72)	16.24 (0.17)	17.34 (0.08)
Avoidable ED visits, percentage points[b]	0.76 (0.42 to 1.09)	13.09 (0.15)	12.33 (0.08)	0.73 (0.38 to 1.07)	12.98 (0.15)	12.25 (0.07)
Preventable hospitalizations, percentage points[b]	-0.10 (-0.33 to 0.14)	5.76 (0.10)	5.85 (0.05)	-0.06 (-0.30 to 0.18)	5.89 (0.11)	5.95 (0.05)
Survival days	-0.68 (-2.65 to 1.28)	430 (0.86)	431 (0.45)	1.73 (-0.25 to 3.71)	433 (0.88)	431 (0.41)

*This is a part of outpatient care

Abbreviations: MA, Medicare Advantage; TM, Traditional Medicare; CI, confidence interval; SE, standard error.

[a] Weighted linear regressions with county fixed effects.

[b] Estimates were multiplied by 100 to show estimates as percentage point differences.

© 2024 Kalidindi Y et al. *JAMA Network Open.*

eTable 4. Regression Results and Adjusted Outcomes: Using 12-Month Chemotherapy Episode

Outcome	Regression results, coefficient (95% CI)[a]	Adjusted means (SE)	
		MA	TM
Resource use, US $			
Total resource use	-13,025 (-13,572 to -12,478)	91,147 (224.87)	104,171 (144.36)
By service type			
Hospital inpatient services	-1,152 (-1,341 to -962)	14,481 (77.02)	15,633 (52.46)
Outpatient care	-12,492 (-12,929 to -12,055)	53,668 (178.35)	66,160 (116.59)
Prescription drugs	538 (212 to 865)	21,780 (134.55)	21,241 (87.59)
Hospice services	81 (44 to 117)	1,218 (15.54)	1,137 (9.32)
Chemotherapy services*			
Chemotherapy services, US $			
Part B chemotherapy	-7,345 (-7,693 to -6,996)	26,151 (139.23)	33,496 (99.00)
Part B chemotherapy- supportive drugs	-596 (-620 to -571)	2,666 (9.72)	3,262 (6.50)
Part D chemotherapy	488 (174 to 802)	17,910 (129.19)	17,421 (84.26)
Sources of differences in part B chemotherapy			
Chemotherapy visits per episode	-1.58 (-1.64 to -1.52)	7.76 (0.02)	9.34 (0.02)
Resource use/chemotherapy visit, US $	-234 (-285 to -183)	3,329 (21.57)	3,563 (9.79)
Quality of care			
Chemotherapy-related ED visits, percentage points[b]	-3.01 (-3.36 to -2.67)	23.4 (0.14)	26.4 (0.10)
Chemotherapy-related hospital admission, percentage points[b]	-1.23 (-1.53 to -0.93)	16.1 (0.12)	17.3 (0.08)
Avoidable ED visits, percentage points[b]	0.89 (0.57 to 1.20)	18.8 (0.13)	17.9 (0.08)
Preventable hospitalizations, percentage points[b]	-0.26 (-0.49 to -0.35)	8.7 (0.10)	9.0 (0.06)
Survival days	1.17 (-0.25 to 2.58)	432 (0.59)	431 (0.38)

*This is a part of outpatient care

Abbreviations: MA, Medicare Advantage; TM, Traditional Medicare; CI, confidence interval; SE, standard error.

[a] Weighted linear regressions with county fixed effects.

[b] Estimates were multiplied by 100 to show estimates as percentage point differences.

© 2024 Kalidindi Y et al. *JAMA Network Open.*

eTable 5. Regression Results and Adjusted Outcomes from Analysis of Using First Chemotherapy Episode

Outcome	Regression results, coefficient (95% CI)[a]	Adjusted means (SE)	
		MA	TM
Resource use, US $			
Total resource use	-8,565 (-8,892 to -8,238)	60,843 (135.69)	69,408 (83.01)
By service type			
Hospital inpatient services	-696 (-826 to -566)	9,283 (52.49)	9,979 (36.95)
Outpatient care	-8,293 (-8,576 to -8,011)	38,397 (116.94)	46,690 (71.18)
Prescription drugs	374 (204 to 545)	12,607 (69.65)	12,233 (46.80)
Hospice services	50 (31 to 69)	556 (7.99)	506 (4.83)
Chemotherapy services*			
Chemotherapy services, US $			
Part B chemotherapy	-4,734 (-4,956 to -4,512)	18,710 (88.64)	23,444 (60.92)
Part B chemotherapy- supportive drugs	-425 (-444 to -405)	2,247 (9.72)	2,671 (5.18)
Part D chemotherapy	309 (146 to 473)	10,370 (66.74)	10,060 (44.79)
Sources of differences in part B chemotherapy			
Chemotherapy visits per episode	-1.03 (-1.07 to -0.99)	5.96 (0.01)	6.99 (0.01)
Resource use/chemotherapy visit, US $	-228 (-274 to -182)	3,333 (19.32)	3,561 (9.43)
Quality of care			
Chemotherapy-related ED visits, percentage points[b]	-2.89 (-3.21 to -2.58)	23.7 (0.13)	26.6 (0.09)
Chemotherapy-related hospital admission, percentage points[b]	-1.15 (-1.43 to -0.87)	16.3 (0.11)	17.4 (0.08)
Avoidable ED visits, percentage points[b]	0.68 (0.43 to 0.93)	13.0 (0.10)	12.3 (0.07)
Preventable hospitalizations, percentage points[b]	-0.11 (-0.28 to 0.07)	5.8 (0.07)	5.9 (0.05)
Survival days	1.19 (-0.24 to 2.62)	432 (0.59)	431 (0.38)

*This is a part of outpatient care

Abbreviations: MA, Medicare Advantage; TM, Traditional Medicare; CI, confidence interval; SE, standard error.

[a] Weighted linear regressions with county fixed effects.

[b] Estimates were multiplied by 100 to show estimates as percentage point differences.

© 2024 Kalidindi Y et al. *JAMA Network Open.*

eTable 6. GLM for Resource Use and Logit for Quality Outcomes

Outcome	Regression results, coefficient (95% CI)[a]	Adjusted means (SE)	
		MA	TM
Resource use, US $			
Total resource use	-8,724 (-9,031 to -8,417)	60,905 (131.39)	69,629 (78.56)
By service type			
Hospital inpatient services	-782 (-911 to -654)	9,265 (51.51)	10,047 (37.42)
Outpatient care	-8,219 (-8,499 to -7,939)	38,409 (115.83)	46,628 (70.25)
Prescription drugs	345 (111 to 579)	13,171 (83.97)	12,826 (64.80)
Hospice services	98 (66 to 130)	663 (12.62)	565 (10.01)
Chemotherapy services*			
Chemotherapy services, US $			
Part B chemotherapy	-4,684 (-4,926 to -4,443)	19,010 (90.08)	23,694 (68.80)
Part B chemotherapy- supportive drugs	-482 (-501 to -464)	2,191 (7.41)	2,673 (5.38)
Part D chemotherapy	577 (225 to 928)	11,396 (112.47)	10,819 (104.11)
Sources of differences in part B chemotherapy			
Chemotherapy visits per episode	-1.00 (-1.04 to -0.97)	5.93 (0.01)	6.93 (0.01)
Resource use/chemotherapy visit, US $	-234 (-276 to -191)	3,383 (17.58)	3,616 (10.55)
Quality of care			
Chemotherapy-related ED visits, percentage points[b]	-2.87 (-3.25 to -2.48)	23.6 (0.19)	26.4 (0.15)
Chemotherapy-related hospital admission, percentage points[b]	-1.28 (-1.57 to -1.00)	16.1 (0.13)	17.4 (0.10)
Avoidable ED visits, percentage points[b]	0.46 (0.19 to 0.73)	12.8 (0.13)	12.3 (0.11)
Preventable hospitalizations, percentage points[b]	-0.17 (-0.34 to 0.00)	5.7 (0.08)	5.9 (0.06)
Survival days	1.68 (0.17 to 3.19)	432 (0.62)	431 (0.38)

*This is a part of outpatient care

Abbreviations: MA, Medicare Advantage; TM, Traditional Medicare; CI, confidence interval; SE, standard error.

[a] Weighted linear regressions with county fixed effects.

[b] Estimates were multiplied by 100 to show estimates as percentage point differences.

© 2024 Kalidindi Y et al. *JAMA Network Open.*

eTable 7. Drug List

The list of Healthcare Common Procedure Coding System (HCPCS) codes used to identify Part B chemotherapy drugs and Part B supportive care drugs.

This list was developed using a combination of chemotherapy codes used in the Oncology Care Model (OCM)[1] and chemotherapy codes identified using the Restructured BETOS Classification System (RBCS).[2]

Type of Part B Care	***HCPCS***	***Description/Generic Drug Name***
Chemotherapy	A9513	LUTETIUM LU 177 DOTATATE
Chemotherapy	A9543	IBRITUMOMAB
Chemotherapy	A9545	TOSITUMOMAB
Chemotherapy	A9590	IOBENGUANE I 131
Chemotherapy	A9606	RADIUM 223
Chemotherapy	C9016	TRIPTORELIN
Chemotherapy	C9021	OBINUTUZUMAB
Chemotherapy	C9024	DAUNORUBICIN AND CYTARABINE
Chemotherapy	C9025	RAMUCIRUMAB
Chemotherapy	C9027	PEMBROLIZUMAB
Chemotherapy	C9028	INOTUZUMAB OZOGAMICIN
Chemotherapy	C9030	COPANLISIB
Chemotherapy	C9031	LUTETIUM LU 177 DOTATATE
Chemotherapy	C9038	MOGAMULIZUMAB
Chemotherapy	C9042	BENDAMUSTINE
Chemotherapy	C9044	CEMIPLIMAB-RWLC
Chemotherapy	C9045	MOXETUMOMAB PASUDOTOX-TDFK
Chemotherapy	C9049	TAGRAXOFUSP-ERZS
Chemotherapy	C9050	EMAPALUMAB
Chemotherapy	C9062	DARATUMUMAB AND HYALURONIDASE-FIHJ
Chemotherapy	C9064	MITOMYCIN
Chemotherapy	C9065	ROMIDEPSIN
Chemotherapy	C9066	SACITUZUMAB GOVITECAN-HZIY
Chemotherapy	C9069	BELANTAMAB MAFODOTIN-BLMF

© 2024 Kalidindi Y et al. *JAMA Network Open.*

Type of Part B Care	*HCPCS*	*Description/Generic Drug Name*
Chemotherapy	C9070	TAFASITAMAB-CXIX
Chemotherapy	C9073	BREXUCABTAGENE AUTOLEUCEL
Chemotherapy	C9131	ADO-TRASTUZUMAB EMTANSINE
Chemotherapy	C9257	BEVACIZUMAB
Chemotherapy	C9287	BRENTUXIMAB VEDOTIN
Chemotherapy	C9289	ASPARAGINASE ERWINIA
Chemotherapy	C9292	PERTUZUMAB
Chemotherapy	C9295	CARFILZOMIB
Chemotherapy	C9296	ZIV-AFLIBERCEPT
Chemotherapy	C9297	OMACETAXINE
Chemotherapy	C9408	IOBENGUANE I 131
Chemotherapy	C9416	BCG
Chemotherapy	C9442	BELINOSTAT
Chemotherapy	C9449	BLINATUMOMAB
Chemotherapy	C9453	NIVOLUMAB
Chemotherapy	C9455	SILTUXIMAB
Chemotherapy	C9467	RITUXIMAB AND HYALURONIDASE
Chemotherapy	C9472	TALIMOGENE LAHERPAREPVEC
Chemotherapy	C9474	IRINOTECAN, LIPOSOMAL
Chemotherapy	C9475	NECITUMUMAB
Chemotherapy	C9476	DARATUMUMAB
Chemotherapy	C9477	ELOTUZUMAB
Chemotherapy	C9480	TRABECTEDIN
Chemotherapy	C9483	ATEZOLIZUMAB
Chemotherapy	C9485	OLARATUMAB
Chemotherapy	C9491	AVELUMAB
Chemotherapy	C9492	DURVALUMAB
Chemotherapy	J0202	ALEMTUZUMAB
Chemotherapy	J0594	BUSULFAN
Chemotherapy	J0640	LEUCOVORIN
Chemotherapy	J0894	DECITABINE

© 2024 Kalidindi Y et al. *JAMA Network Open.*

Type of Part B Care	*HCPCS*	*Description/Generic Drug Name*
Chemotherapy	J1100	DEXAMETHASONE
Chemotherapy	J1675	HISTRELIN
Chemotherapy	J1930	LANREOTIDE
Chemotherapy	J1950	LEUPROLIDE
Chemotherapy	J2353	OCTREOTIDE
Chemotherapy	J2354	OCTREOTIDE
Chemotherapy	J2860	SILTUXIMAB
Chemotherapy	J2920	METHYLPREDNISOLONE
Chemotherapy	J2930	METHYLPREDNISOLONE
Chemotherapy	J3315	TRIPTORELIN
Chemotherapy	J3316	TRIPTORELIN
Chemotherapy	J7504	ANTI-THYMOCYTE GLOBULIN, EQUINE
Chemotherapy	J7509	METHYLPREDNISOLONE
Chemotherapy	J7511	ANTI-THYMOCYTE GLOBULIN, RABBIT
Chemotherapy	J7520	SIROLIMUS
Chemotherapy	J7527	EVEROLIMUS
Chemotherapy	J8501	APREPITANT
Chemotherapy	J8510	BUSULFAN
Chemotherapy	J8515	CABERGOLINE
Chemotherapy	J8520	CAPECITABINE
Chemotherapy	J8521	CAPECITABINE
Chemotherapy	J8530	CYCLOPHOSPHAMIDE
Chemotherapy	J8540	DEXAMETHASONE
Chemotherapy	J8560	ETOPOSIDE
Chemotherapy	J8561	EVEROLIMUS
Chemotherapy	J8562	FLUDARABINE
Chemotherapy	J8565	GEFITINIB
Chemotherapy	J8597	ANTIEMETIC DRUG, NOT SPECIFIED
Chemotherapy	J8600	MELPHALAN
Chemotherapy	J8610	METHOTREXATE
Chemotherapy	J8650	NABILONE

© 2024 Kalidindi Y et al. *JAMA Network Open.*

Type of Part B Care	*HCPCS*	*Description/Generic Drug Name*
Chemotherapy	J8655	NETUPITANT
Chemotherapy	J8670	ROLAPITANT
Chemotherapy	J8700	TEMOZOLOMIDE
Chemotherapy	J8705	TOPOTECAN
Chemotherapy	J8999	ANTINEO, NOC
Chemotherapy	J9000	DOXORUBICIN
Chemotherapy	J9001	DOXORUBICIN, LIPOSOMAL
Chemotherapy	J9002	DOXORUBICIN, LIPOSOMAL
Chemotherapy	J9010	ALEMTUZUMAB
Chemotherapy	J9015	ALDESLEUKIN
Chemotherapy	J9017	ARSENIC TRIOXIDE
Chemotherapy	J9019	ASPARAGINASE ERWINIA
Chemotherapy	J9020	ASPARAGINASE
Chemotherapy	J9021	ASPARAGINASE
Chemotherapy	J9022	ATEZOLIZUMAB
Chemotherapy	J9023	AVELUMAB
Chemotherapy	J9025	AZACITIDINE
Chemotherapy	J9027	CLOFARABINE
Chemotherapy	J9030	BCG (BACILLUS CALMETTE-GUERIN)
Chemotherapy	J9031	BCG (BACILLUS CALMETTE-GUERIN)
Chemotherapy	J9032	BELINOSTAT
Chemotherapy	J9033	BENDAMUSTINE
Chemotherapy	J9034	BENDAMUSTINE
Chemotherapy	J9035	BEVACIZUMAB
Chemotherapy	J9036	BENDAMUSTINE
Chemotherapy	J9037	BELANTAMAB MAFODOTIN-BLMF
Chemotherapy	J9039	BLINATUMOMAB
Chemotherapy	J9040	BLEOMYCIN
Chemotherapy	J9041	BORTEZOMIB
Chemotherapy	J9042	BRENTUXIMAB VEDOTIN
Chemotherapy	J9043	CABAZITAXEL

© 2024 Kalidindi Y et al. *JAMA Network Open.*

Type of Part B Care	*HCPCS*	*Description/Generic Drug Name*
Chemotherapy	J9044	BORTEZOMIB
Chemotherapy	J9045	CARBOPLATIN
Chemotherapy	J9047	CARFILZOMIB
Chemotherapy	J9050	CARMUSTINE
Chemotherapy	J9055	CETUXIMAB
Chemotherapy	J9057	COPANLISIB
Chemotherapy	J9060	CISPLATIN
Chemotherapy	J9062	CISPLATIN
Chemotherapy	J9065	CLADRIBINE
Chemotherapy	J9070	CYCLOPHOSPHAMIDE
Chemotherapy	J9080	CYCLOPHOSPHAMIDE
Chemotherapy	J9090	CYCLOPHOSPHAMIDE
Chemotherapy	J9091	CYCLOPHOSPHAMIDE
Chemotherapy	J9092	CYCLOPHOSPHAMIDE
Chemotherapy	J9093	CYCLOPHOSPHAMIDE
Chemotherapy	J9094	CYCLOPHOSPHAMIDE
Chemotherapy	J9095	CYCLOPHOSPHAMIDE
Chemotherapy	J9096	CYCLOPHOSPHAMIDE
Chemotherapy	J9097	CYCLOPHOSPHAMIDE
Chemotherapy	J9098	CYTARABINE, LIPOSOMAL
Chemotherapy	J9100	CYTARABINE
Chemotherapy	J9110	CYTARABINE
Chemotherapy	J9118	CALASPARGASE PEGOL-MKNL
Chemotherapy	J9119	CEMIPLIMAB-RWLC
Chemotherapy	J9120	DACTINOMYCIN
Chemotherapy	J9130	DACARBAZINE
Chemotherapy	J9140	DACARBAZINE
Chemotherapy	J9144	DARATUMUMAB AND HYALURONIDASE-FIHJ
Chemotherapy	J9145	DARATUMUMAB
Chemotherapy	J9150	DAUNORUBICIN
Chemotherapy	J9151	DAUNORUBICIN, LIPOSOMAL

Type of Part B Care	*HCPCS*	*Description/Generic Drug Name*
Chemotherapy	J9153	DAUNORUBICIN AND CYTARABINE
Chemotherapy	J9155	DEGARELIX
Chemotherapy	J9160	DENILEUKIN DIFTITOX
Chemotherapy	J9165	DIETHYLSTILBESTROL DIPHOSPHATE
Chemotherapy	J9170	DOCETAXEL
Chemotherapy	J9171	DOCETAXEL
Chemotherapy	J9173	DURVALUMAB
Chemotherapy	J9175	ELLIOTS' B
Chemotherapy	J9176	ELOTUZUMAB
Chemotherapy	J9177	ENFORTUMAB VEDOTIN-EJFV
Chemotherapy	J9178	EPIRUBICIN
Chemotherapy	J9179	ERIBULIN
Chemotherapy	J9180	EPIRUBICIN
Chemotherapy	J9181	ETOPOSIDE
Chemotherapy	J9182	ETOPOSIDE
Chemotherapy	J9185	FLUDARABINE
Chemotherapy	J9190	FLUOROURACIL
Chemotherapy	J9198	GEMCITABINE
Chemotherapy	J9199	GEMCITABINE
Chemotherapy	J9200	FLOXURIDINE
Chemotherapy	J9201	GEMCITABINE
Chemotherapy	J9202	GOSERELIN
Chemotherapy	J9203	GEMTUZUMAB OZOGAMICIN
Chemotherapy	J9204	MOGAMULIZUMAB
Chemotherapy	J9205	IRINOTECAN, LIPOSOMAL
Chemotherapy	J9206	IRINOTECAN
Chemotherapy	J9207	IXABEPILONE
Chemotherapy	J9208	IFOSFAMIDE
Chemotherapy	J9209	MESNA
Chemotherapy	J9210	EMAPALUMAB
Chemotherapy	J9211	IDARUBICIN

Type of Part B Care	*HCPCS*	*Description/Generic Drug Name*
Chemotherapy	J9212	INTERFERON ALFACON
Chemotherapy	J9213	INTERFERON, ALFA-2A
Chemotherapy	J9214	INTERFERON ALFA-2B
Chemotherapy	J9215	INTERFERON, ALFA-N3
Chemotherapy	J9216	INTERFERON, GAMMA 1-B
Chemotherapy	J9217	LEUPROLIDE
Chemotherapy	J9218	LEUPROLIDE
Chemotherapy	J9219	LEUPROLIDE
Chemotherapy	J9223	LURBINECTEDIN
Chemotherapy	J9225	HISTRELIN
Chemotherapy	J9226	HISTRELIN
Chemotherapy	J9227	ISATUXIMAB-IRFC
Chemotherapy	J9228	IPILIMUMAB
Chemotherapy	J9229	INOTUZUMAB OZOGAMICIN
Chemotherapy	J9230	MECHLORETHAMINE
Chemotherapy	J9240	MEDROYPROGESTERONE ACETATE
Chemotherapy	J9245	MELPHALAN
Chemotherapy	J9246	MELPHALAN
Chemotherapy	J9250	METHOTREXATE
Chemotherapy	J9260	METHOTREXATE
Chemotherapy	J9261	NELARABINE
Chemotherapy	J9262	OMACETAXINE
Chemotherapy	J9263	OXALIPLATIN
Chemotherapy	J9264	PACLITAXEL, PROTEIN-BOUND
Chemotherapy	J9265	PACLITAXEL
Chemotherapy	J9266	PEGASPARGASE
Chemotherapy	J9267	PACLITAXEL
Chemotherapy	J9268	PENTOSTATIN
Chemotherapy	J9269	TAGRAXOFUSP-ERZS
Chemotherapy	J9270	PLICAMYCIN
Chemotherapy	J9271	PEMBROLIZUMAB

© 2024 Kalidindi Y et al. *JAMA Network Open.*

Type of Part B Care	*HCPCS*	*Description/Generic Drug Name*
Chemotherapy	J9272	DOSTARLIMAB
Chemotherapy	J9280	MITOMYCIN
Chemotherapy	J9281	MITOMYCIN
Chemotherapy	J9285	OLARATUMAB
Chemotherapy	J9290	MITOMYCIN
Chemotherapy	J9291	MITOMYCIN
Chemotherapy	J9293	MITOXANTRONE
Chemotherapy	J9295	NECITUMUMAB
Chemotherapy	J9299	NIVOLUMAB
Chemotherapy	J9300	GEMTUZUMAB OZOGAMICIN
Chemotherapy	J9301	OBINUTUZUMAB
Chemotherapy	J9302	OFATUMUMAB
Chemotherapy	J9303	PANITUMUMAB
Chemotherapy	J9304	PEMETREXED
Chemotherapy	J9305	PEMETREXED
Chemotherapy	J9306	PERTUZUMAB
Chemotherapy	J9307	PRALATREXATE
Chemotherapy	J9308	RAMUCIRUMAB
Chemotherapy	J9309	POLATUZUMAB VEDOTIN-PIIQ
Chemotherapy	J9310	RITUXIMAB
Chemotherapy	J9311	RITUXIMAB AND HYALURONIDASE
Chemotherapy	J9312	RITUXIMAB
Chemotherapy	J9313	MOXETUMOMAB PASUDOTOX-TDFK
Chemotherapy	J9315	ROMIDEPSIN
Chemotherapy	J9316	PERTUZUMAB, TRASTUZUMAB, AND HYALURONIDASE-ZZXF
Chemotherapy	J9317	SACITUZUMAB GOVITECAN-HZIY
Chemotherapy	J9318	ROMIDEPSIN
Chemotherapy	J9319	ROMIDEPSIN
Chemotherapy	J9320	STREPTOZOCIN
Chemotherapy	J9325	TALIMOGENE LAHERPAREPVEC

© 2024 Kalidindi Y et al. *JAMA Network Open.*

Type of Part B Care	*HCPCS*	*Description/Generic Drug Name*
Chemotherapy	J9328	TEMOZOLOMIDE
Chemotherapy	J9330	TEMSIROLIMUS
Chemotherapy	J9340	THIOTEPA
Chemotherapy	J9348	NAXITAMAB
Chemotherapy	J9349	TAFASITAMAB-CXIX
Chemotherapy	J9350	TOPOTECAN
Chemotherapy	J9351	TOPOTECAN
Chemotherapy	J9352	TRABECTEDIN
Chemotherapy	J9353	MARGETUXIMAB
Chemotherapy	J9354	ADO-TRASTUZUMAB EMTANSINE
Chemotherapy	J9355	TRASTUZUMAB
Chemotherapy	J9356	TRASTUZUMAB AND HYALURONIDASE-OYSK
Chemotherapy	J9357	VALRUBICIN
Chemotherapy	J9358	FAM-TRASTUZUMAB DERUXTECAN-NXKI
Chemotherapy	J9360	VINBLASTINE
Chemotherapy	J9370	VINCRISTINE
Chemotherapy	J9371	VINCRISTINE, LIPOSOMAL
Chemotherapy	J9375	VINCRISTINE
Chemotherapy	J9380	VINCRISTINE
Chemotherapy	J9390	VINORELBINE
Chemotherapy	J9395	FULVESTRANT
Chemotherapy	J9400	ZIV-AFLIBERCEPT
Chemotherapy	J9600	PORFIMER SODIUM
Chemotherapy	J9999	ANTINEOPLASTIC, NOC
Chemotherapy	Q2017	TENIPOSIDE
Chemotherapy	Q2040	TISAGENLECLEUCEL
Chemotherapy	Q2041	AXICABTAGENE CILOLEUCEL
Chemotherapy	Q2042	TISAGENLECLEUCEL
Chemotherapy	Q2043	SIPULEUCEL-T
Chemotherapy	Q2048	DOXORUBICIN, LIPOSOMAL
Chemotherapy	Q2049	DOXORUBICIN, LIPOSOMAL

© 2024 Kalidindi Y et al. *JAMA Network Open.*

Type of Part B Care	*HCPCS*	*Description/Generic Drug Name*
Chemotherapy	Q2050	DOXORUBICIN, LIPOSOMAL
Chemotherapy	Q2053	BREXUCABTAGENE AUTOLEUCEL
Chemotherapy	Q5107	BEVACIZUMAB-AWWB
Chemotherapy	Q5112	TRASTUZUMAB-DTTB
Chemotherapy	Q5113	TRASTUZUMAB-PKRB
Chemotherapy	Q5114	TRASTUZUMAB-DKST
Chemotherapy	Q5115	RITUXIMAB-ABBS
Chemotherapy	Q5116	TRASTUZUMAB-QYYP
Chemotherapy	Q5117	TRASTUZUMAB-ANNS
Chemotherapy	Q5118	BEVACIZUMAB-BVZR
Chemotherapy	Q5119	RITUXIMAB-PVVR
Chemotherapy	Q5123	RITUXIMAB
Chemotherapy	Q9979	ALEMTUZUMAB
Chemotherapy	WW002	TEMOZOLOMIDE
Chemotherapy	WW003	TEMOZOLOMIDE
Chemotherapy	WW004	TEMOZOLOMIDE
Chemotherapy	WW005	TEMOZOLOMIDE
Chemotherapy	WW006	TEMOZOLOMIDE
Chemotherapy	WW007	TEMOZOLOMIDE
Chemotherapy	WW008	TEMOZOLOMIDE
Chemotherapy	WW009	TEMOZOLOMIDE
Chemotherapy	WW020	BUSULFAN
Chemotherapy	WW030	ETOPOSIDE
Chemotherapy	WW031	ETOPOSIDE
Chemotherapy	WW032	ETOPOSIDE
Chemotherapy	WW080	MELPHALAN
Chemotherapy	WW081	MELPHALAN
Chemotherapy	WW089	CAPECITABINE
Chemotherapy	WW090	CAPECITABINE
Chemotherapy	WW091	CAPECITABINE
Chemotherapy	WW093	CAPECITABINE

© 2024 Kalidindi Y et al. *JAMA Network Open.*

Type of Part B Care	*HCPCS*	*Description/Generic Drug Name*
Chemotherapy	WW094	CAPECITABINE
Chemotherapy	WW096	CAPECITABINE
Chemotherapy	WW140	TOPOTECAN
Supportive Care	51720	TREATMENT OF BLADDER LESION
Supportive Care	96401	CHEMO ANTI-NEOPL SQ/IM
Supportive Care	96402	CHEMO HORMON ANTINEOPL SQ/IM
Supportive Care	96405	CHEMO INTRALESIONAL UP TO 7
Supportive Care	96406	CHEMO INTRALESIONAL OVER 7
Supportive Care	96409	CHEMO IV PUSH SNGL DRUG
Supportive Care	96411	CHEMO IV PUSH ADDL DRUG
Supportive Care	96413	CHEMO IV INFUSION 1 HR
Supportive Care	96415	CHEMO IV INFUSION ADDL HR
Supportive Care	96416	CHEMO PROLONG INFUSE W/PUMP
Supportive Care	96417	CHEMO IV INFUS EACH ADDL SEQ
Supportive Care	96420	CHEMO IA PUSH TECNIQUE
Supportive Care	96422	CHEMO IA INFUSION UP TO 1 HR
Supportive Care	96423	CHEMO IA INFUSE EACH ADDL HR
Supportive Care	96425	CHEMOTHERAPY INFUSION METHOD
Supportive Care	96440	CHEMOTHERAPY INTRACAVITARY
Supportive Care	96446	CHEMOTX ADMN PRTL CAVITY
Supportive Care	96450	CHEMOTHERAPY INTO CNS
Supportive Care	96521	REFILL/MAINT PORTABLE PUMP
Supportive Care	96522	REFILL/MAINT PUMP/RESVR SYST
Supportive Care	96523	IRRIG DRUG DELIVERY DEVICE
Supportive Care	96542	CHEMOTHERAPY INJECTION
Supportive Care	96549	UNSPECIFIED CHEMOTHERAPY PROCEDURE
Supportive Care	0540T	CHIMERIC ANTIGEN RECEPTOR T–CELL (CAR–T) THERAPY
Supportive Care	C9033	INJECTION, FOSNETUPITANT 235 MG AND PALONOSETRON 0.25 MG
Supportive Care	C9293	INJECTION, GLUCARPIDASE, 10 UNITS

Type of Part B Care	*HCPCS*	*Description/Generic Drug Name*
Supportive Care	C9448	NETUPITANT 300MG AND PALONOSETRON 0.5 MG, ORAL
Supportive Care	C9463	INJECTION, APREPITANT, 1 MG
Supportive Care	C9464	INJECTION, ROLAPITANT, 0.5 MG
Supportive Care	C9486	INJECTION, GRANISETRON EXTENDED RELEASE, 0.1 MG
Supportive Care	G0498	CHEMOTHERAPY ADMINISTRATION, INTRAVENOUS INFUSION TECHNIQUE
Supportive Care	J0207	INJECTION, AMIFOSTINE, 500 MG
Supportive Care	J0780	INJECTION, PROCHLORPERAZINE, UP TO 10 MG
Supportive Care	J1453	INJECTION, FOSAPREPITANT, 1 MG
Supportive Care	J1454	INJECTION, FOSNETUPITANT 235 MG AND PALONOSETRON 0.25 MG
Supportive Care	J1626	INJECTION, GRANISETRON HYDROCHLORIDE, 100 MCG
Supportive Care	J1627	INJECTION, GRANISETRON, EXTENDED-RELEASE, 0.1 MG
Supportive Care	J1790	INJECTION, DROPERIDOL, UP TO 5 MG
Supportive Care	J2469	INJECTION, PALONOSETRON HCL, 25 MCG
Supportive Care	J2797	INJECTION, ROLAPITANT, 0.5 MG
Supportive Care	J3250	INJECTION, TRIMETHOBENZAMIDE HCL, UP TO 200 MG
Supportive Care	J7503	TACROLIMUS, EXTENDED RELEASE, (ENVARSUS XR), ORAL, 0.25 MG
Supportive Care	J7505	MUROMONAB-CD3, PARENTERAL, 5 MG
Supportive Care	Q0083	CHEMOTHERAPY ADMINISTRATION BY OTHER THAN INFUSION
Supportive Care	Q0084	CHEMOTHERAPY ADMINISTRATION BY INFUSION TECHNIQUE
Supportive Care	Q0085	CHEMOTHERAPY ADMINISTRATION BY BOTH INFUSION TECHNIQUE AND OTHER TECHNIQUE
Supportive Care	Q0161	CHLORPROMAZINE HYDROCHLORIDE, 5 MG, ORAL
Supportive Care	Q0162	ONDANSETRON 1 MG, ORAL
Supportive Care	Q0163	DIPHENHYDRAMINE HYDROCHLORIDE, 50 MG, ORAL
Supportive Care	Q0164	PROCHLORPERAZINE MALEATE, 5 MG, ORAL
Supportive Care	Q0166	GRANISETRON HYDROCHLORIDE, 1 MG, ORAL
Supportive Care	Q0167	DRONABINOL, 2.5 MG, ORAL

© 2024 Kalidindi Y et al. *JAMA Network Open.*

Type of Part B Care	*HCPCS*	*Description/Generic Drug Name*
Supportive Care	Q0169	PROMETHAZINE HYDROCHLORIDE, 12.5 MG, ORAL
Supportive Care	Q0173	TRIMETHOBENZAMIDE HYDROCHLORIDE, 250 MG, ORAL
Supportive Care	Q0175	PERPHENAZINE, 4 MG, ORAL
Supportive Care	Q0177	HYDROXYZINE PAMOATE, 25 MG, ORAL
Supportive Care	Q0180	DOLASETRON MESYLATE, 100 MG, ORAL
Supportive Care	Q0181	UNSPECIFIED ORAL DOSAGE FORM, ANTI-EMETIC
Supportive Care	Q0510	PHARMACY SUPPLY FEE
Supportive Care	Q0511	PHARMACY SUPPLY FEE
Supportive Care	Q0512	PHARMACY SUPPLY FEE
Supportive Care	Q2052	SERVICES, SUPPLIES AND ACCESSORIES, IVIG DEMO
Supportive Care	Q5111	INJECTION, PEGFILGRASTIM-CBQV (UDENYCA), BIOSIMILAR, 0.5 MG
Supportive Care	Q9978	NETUPITANT 300 MG AND PALONOSETRON 0.5 MG, ORAL
Supportive Care	Q9981	ROLAPITANT, ORAL, 1 MG

© 2024 Kalidindi Y et al. *JAMA Network Open.*

Part D chemotherapy drugs were identified using the NDC list available in the OCM list of initiating therapies.[1] The generic drugs included are listed below.

Generic Drug Name
ABEMACICLIB
ABIRATERONE
ACALABRUTINIB
ADO-TRASTUZUMAB EMTANSINE
AFATINIB
ALDESLEUKIN
ALECTINIB
ALEMTUZUMAB
ALPELISIB
ALTRETAMINE
ANASTROZOLE
ANTI-THYMOCYTE GLOBULIN, EQUINE
ANTI-THYMOCYTE GLOBULIN, RABBIT
APALUTAMIDE
ARSENIC TRIOXIDE
ASPARAGINASE
ATEZOLIZUMAB
AVAPRITINIB
AVELUMAB
AXICABTAGENE CILOLEUCEL
AXITINIB
AZACITIDINE
BCG (BACILLUS CALMETTE-GUERIN)
BELANTAMAB MAFODOTIN-BLMF
BELINOSTAT
BENDAMUSTINE
BENDAMUSTINE
BEVACIZUMAB

© 2024 Kalidindi Y et al. *JAMA Network Open.*

Generic Drug Name
BEVACIZUMAB-AWWB
BEVACIZUMAB-BVZR
BEXAROTENE
BICALUTAMIDE
BINIMETINIB
BLEOMYCIN
BLINATUMOMAB
BORTEZOMIB
BOSUTINIB
BOSUTINIB
BRENTUXIMAB VEDOTIN
BREXUCABTAGENE AUTOLEUCEL
BRIGATINIB
BUSULFAN
CABAZITAXEL
CABOZANTINIB
CABOZANTINIB
CALASPARGASE PEGOL-MKNL
CAPECITABINE
CAPMATINIB
CARBOPLATIN
CARFILZOMIB
CARMUSTINE
CEMIPLIMAB-RWLC
CERITINIB
CETUXIMAB
CHLORAMBUCIL
CISPLATIN
CLADRIBINE
CLOFARABINE
COBIMETINIB

© 2024 Kalidindi Y et al. *JAMA Network Open.*

Generic Drug Name
COPANLISIB
CRIZOTINIB
CYCLOPHOSPHAMIDE
CYTARABINE
CYTARABINE, LIPOSOMAL
DABRAFENIB
DACARBAZINE
DACOMITINIB
DACTINOMYCIN
DARATUMUMAB
DARATUMUMAB AND HYALURONIDASE-FIHJ
DAROLUTAMIDE
DASATINIB
DAUNORUBICIN
DAUNORUBICIN
DAUNORUBICIN AND CYTARABINE
DAUNORUBICIN, LIPOSOMAL
DECITABINE
DECITABINE AND CEDAZURIDINE
DEGARELIX
DENILEUKIN DIFTITOX
DINUTUXIMAB
DOCETAXEL
DOXORUBICIN
DOXORUBICIN, LIPOSOMAL
DURVALUMAB
DUVELISIB
ELOTUZUMAB
ENASIDENIB
ENCORAFENIB
ENFORTUMAB VEDOTIN-EJFV

© 2024 Kalidindi Y et al. *JAMA Network Open.*

Generic Drug Name
ENTRECTINIB
ENZALUTAMIDE
EPIRUBICIN
ERDAFITINIB
ERIBULIN
ERLOTINIB
ESTRAMUSTINE
ETOPOSIDE
EVEROLIMUS
EXEMESTANE
FAM-TRASTUZUMAB DERUXTECAN-NXKI
FEDRATINIB
FLOXURIDINE
FLUDARABINE
FLUOROURACIL
FLUTAMIDE
FULVESTRANT
GEFITINIB
GEMCITABINE
GEMTUZUMAB OZOGAMICIN
GILTERITINIB
GLASDEGIB
GOSERELIN
HISTRELIN
IBRITUMOMAB
IBRUTINIB
IDARUBICIN
IDELALISIB
IFOSFAMIDE
IMATINIB
INOTUZUMAB OZOGAMICIN

© 2024 Kalidindi Y et al. *JAMA Network Open.*

Generic Drug Name
INTERFERON, GAMMA 1-B
IOBENGUANE I 131
IPILIMUMAB
IRINOTECAN
IRINOTECAN, LIPOSOMAL
ISATUXIMAB-IRFC
IVOSIDENIB
IXABEPILONE
IXAZOMIB
LANREOTIDE
LAPATINIB
LAROTRECTINIB
LENALIDOMIDE
LENVATINIB
LETROZOLE
LETROZOLE AND RIBOCICLIB
LEUPROLIDE
LISOCABTAGENE MARALEUCEL
LOMUSTINE
LORLATINIB
LURBINECTEDIN
LUTETIUM LU 177 DOTATATE
MECHLORETHAMINE
MELPHALAN
MELPHALAN FLUFENAMIDE
MIDOSTAURIN
MITOMYCIN
MITOTANE
MITOXANTRONE
MOXETUMOMAB PASUDOTOX-TDFK
NECITUMUMAB

© 2024 Kalidindi Y et al. *JAMA Network Open.*

Generic Drug Name
NELARABINE
NERATINIB
NILOTINIB
NILOTINIB
NILUTAMIDE
NIRAPARIB
NIVOLUMAB
OBINUTUZUMAB
OCTREOTIDE
OFATUMUMAB
OLAPARIB
OMACETAXINE
OSIMERTINIB
OXALIPLATIN
PACLITAXEL
PACLITAXEL, PROTEIN-BOUND
PALBOCICLIB
PANITUMUMAB
PANOBINOSTAT
PAZOPANIB
PEGASPARGASE
PEMBROLIZUMAB
PEMETREXED
PEMIGATINIB
PENTOSTATIN
PERTUZUMAB
PERTUZUMAB, TRASTUZUMAB, AND HYALURONIDASE-ZZXF
PEXIDARTINIB
POLATUZUMAB
POLATUZUMAB VEDOTIN-PIIQ

© 2024 Kalidindi Y et al. *JAMA Network Open.*

Generic Drug Name
POMALIDOMIDE
PONATINIB
PRALATREXATE
PRALSETINIB
PROCARBAZINE
RAMUCIRUMAB
REGORAFENIB
RELUGOLIX
RIBOCICLIB
RIPRETINIB
RITUXIMAB
RITUXIMAB AND HYALURONIDASE
RITUXIMAB-ABBS
RITUXIMAB-ARRX
RITUXIMAB-PVVR
ROMIDEPSIN
RUCAPARIB
RUXOLITINIB
SACITUZUMAB GOVITECAN-HZIY
SELINEXOR
SELPERCATINIB
SILTUXIMAB
SIPULEUCEL-T
SONIDEGIB
SORAFENIB
STREPTOZOCIN
SUNITINIB
TAFASITAMAB-CXIX
TAGRAXOFUSP-ERZS
TALAZOPARIB
TALIMOGENE LAHERPAREPVEC

© 2024 Kalidindi Y et al. *JAMA Network Open.*

Generic Drug Name
TAMOXIFEN
TAZEMETOSTAT
TEMOZOLOMIDE
TEMSIROLIMUS
TENIPOSIDE
TEPOTINIB
THALIDOMIDE
THIOGUANINE
THIOTEPA
TISAGENLECLEUCEL
TIVOZANIB
TOPOTECAN
TOREMIFENE
TOSITUMOMAB
TRABECTEDIN
TRAMETINIB
TRASTUZUMAB
TRASTUZUMAB AND HYALURONIDASE-OYSK
TRASTUZUMAB-ANNS
TRASTUZUMAB-DKST
TRASTUZUMAB-DTTB
TRASTUZUMAB-PKRB
TRASTUZUMAB-QYYP
TRIFLURIDINE/TIPIRACIL
TRIPTORELIN
TRIPTORELIN
TUCATINIB
UMBRALISIB
VALRUBICIN
VANDETANIB
VEMURAFENIB

© 2024 Kalidindi Y et al. *JAMA Network Open.*

Generic Drug Name
VENETOCLAX
VINBLASTINE
VINCRISTINE
VINCRISTINE, LIPOSOMAL
VINORELBINE
VISMODEGIB
VORINOSTAT
ZANUBRUTINIB
ZIV-AFLIBERCEPT

References:

1) Centers for Medicare & Medicaid Services. Oncology Care Model Performance-Based Payment Methodology Version 7.1. Centers for Medicare & Medicaid Services (CMS); 2021. Accessed April 12, 2024. https://www.cms.gov/priorities/innovation/files/x/ocm-pp3beyond-pymmeth.pd
2) Centers for Medicare & Medicaid Services. Restructured BETOS Classification System; 2021. Accessed February 29, 2024. https://data.cms.gov/provider-summary-by-type-of-service/provider-service-classifications/restructured-betos-classification-system
