# Supplementary material for: Resource Use and Care Quality Differences Among Medicare Beneficiaries Undergoing Chemotherapy
Source: JAMA Netw Open. 2024 Sep 20;7(9):e2434707. doi: 10.1001/jamanetworkopen.2024.34707 (PMC11415781; doi:10.1001/jamanetworkopen.2024.34707)
Supplement: Supplement 2. — Data Sharing Statement [file jamanetwopen-e2434707-s002.pdf]

Data Sharing Statement

Kalidindi. Resource Use and Care Quality Differences Among Medicare Beneficiaries Undergoing Chemotherapy. *JAMA Netw Open*. Published September 20, 2024. doi:10.1001/jamanetworkopen.2024.34707

Data
Data available: No

Additional Information
Explanation for why data not available: RIF Medicare Claims Data Analysis
